# Immunophenotype associated with high sustained antibody titers against enzyme replacement therapy in infantile-onset Pompe disease

**DOI:** 10.3389/fimmu.2023.1301912

**Published:** 2024-01-04

**Authors:** Ankit K. Desai, P. Brian Smith, John S. Yi, Amy S. Rosenberg, Trevor D. Burt, Priya S. Kishnani

**Affiliations:** ^1^ Division of Medical Genetics, Department of Pediatrics, Duke University Health System, Durham, NC, United States; ^2^ Division of Neonatology, Department of Pediatrics, Duke University School of Medicine, Durham, NC, United States; ^3^ Duke Clinical Research Institute, Durham, NC, United States; ^4^ Division of Surgical Sciences, Department of Surgery, Duke University School of Medicine, Durham, NC, United States; ^5^ EpiVax, Inc., Providence, RI, United States; ^6^ Children’s Health and Discovery Initiative, Duke University School of Medicine, Durham, NC, United States

**Keywords:** infantile-onset Pompe disease, immunophenotyping, anti-drug antibodies, immune profiling, immune activation, enzyme replacement therapy

## Abstract

**Introduction:**

The efficacy of enzyme replacement therapy (ERT) with alglucosidase alfa for infantile-onset Pompe disease (IOPD) is limited in some patients due to the development of high and sustained antibody titers (HSAT; ≥12,800).

**Methods:**

We carried out detailed immunophenotyping of IOPD patients (n=40), including analysis of circulating cell populations by flow cytometry and plasma cytokines by multiplex array, to determine whether patients with HSAT have unique immunological characteristics compared to those with low titers (LT; <12,800).

**Results:**

Compared to patients with LT, patients who develop HSAT were skewed toward a type 2 immune profile, with an increased frequency of Th2 cells that was positively correlated with levels of Th2 (IL-4, IL-5, IL-13) and pro-inflammatory (IL-6, TNF-α, MIP-1α, MIP-1β) cytokines. B cells were increased in HSAT patients with a decreased fraction of unswitched memory B cells. Plasma GM-CSF concentrations were lower on average in HSAT patients, while CXCL11 was elevated. Finally, using principal components analysis, we derived an HSAT Signature Score that successfully stratified patients according to their antibody titers.

**Discussion:**

The immune profiles revealed in this study not only identify potential biomarkers of patients that developed HSAT but also provide insights into the pathophysiology of HSAT that will ultimately lead to improved immunotherapy strategies.

## 1 Introduction

Pompe disease (OMIM 232300, glycogen storage disease II) is an autosomal recessive disorder characterized by deficiency of acid α-glucosidase (GAA), an enzyme required for the breakdown of lysosomal glycogen (1). Based on the age of symptom onset and presence or absence of cardiomyopathy, Pompe disease is classified into two major subtypes, infantile-onset Pompe disease (IOPD) and late-onset Pompe disease (LOPD). Patients with IOPD have minimal to no residual enzyme activity, which results in very early and rapid glycogen accumulation in skeletal, cardiac, and smooth muscle. This glycogen accumulation leads to hypotonia, cardiomyopathy, and respiratory failure within days to weeks after birth, progressing to death by two years of age (2). In contrast, patients with LOPD have slower disease progression and may present from the first year to the sixth decade of life (3, 4).

In 2006, recombinant human acid α-glucosidase (rhGAA; alglucosidase alfa) was FDA approved as the first therapy for Pompe disease. The availability of enzyme replacement therapy (ERT) with rhGAA has increased the life span and improved the quality of life for patients with IOPD (5, 6). While such improvements have benefited patients with Pompe disease overall, there is marked individual variability in treatment response to ERT and identifying patients who are likely to have a poor response to ERT remains a challenge. The response to ERT can be influenced by various factors such as age at initiation of ERT, angiotensin-converting enzyme (ACE) genotype, extent of preexisting pathology, cross-reactive immunologic material (CRIM) status, and, critically, the development of anti-rhGAA IgG antibodies (7–11).

CRIM-negative status and development of high and sustained anti-rhGAA IgG antibody titers (HSAT; defined as antibody titers ≥12,800), were identified as poor prognostic factors for patients with IOPD on ERT (7, 10, 12). CRIM status (positive or negative) is defined as the presence or absence of GAA protein expressed by the patient (13). CRIM-negative IOPD patients have two severe *GAA* variants and do not make any native GAA enzyme, leaving them prone to development of anti-drug antibodies to ERT due to their lack of natural immune tolerance, as GAA would normally be treated by the immune system as a self-antigen. As such, their immune systems recognize rhGAA as fully foreign, leading to development of HSAT against ERT. In contrast, CRIM-positive IOPD patients express some residual GAA enzyme that allows for generation of natural immune tolerance to some extent.

To minimize the deleterious effects of anti-rhGAA IgG antibodies, various immune modulation strategies have been tried over the years. Among these approaches, a protocol of immune tolerance induction (ITI) with a short-course of rituximab, methotrexate, and IVIG has proven to be the most effective when initiated in ERT-naïve settings, and this has become a standard of care in CRIM-negative IOPD patients to block adaptive immune responses (14, 15). Widespread use of this ITI protocol, especially in high-risk CRIM-negative IOPD patients, has led to increased survival. While CRIM-negative status is a clear risk factor for HSAT and an indicator for ITI therapy, CRIM-positive status is not always predictive of a low anti-rhGAA IgG antibody response as approximately 32% of patients with CRIM-positive IOPD, and a subset of LOPD, patients develop HSAT resulting in a poor clinical response to treatment (10). This highlights the significant need for an immune biomarker or signature that could identify high-risk CRIM-positive patients at diagnosis before starting ERT and/or identify signs of sensitization during the course of therapy. Development of such a screening and monitoring tool is currently hampered by our limited understanding of the immunobiology that leads to HSAT against ERT in Pompe disease.

To address the critical unmet need of elucidating the cellular subsets and cytokine mediators that drive the HSAT response, we carried out multiparameter immunophenotyping by flow cytometry of peripheral blood mononuclear cell (PBMC) populations combined with a multiplex array analysis of plasma cytokine levels in a cohort of deeply phenotyped CRIM-positive and CRIM-negative IOPD patients. Development of a specific, sustained antibody response requires not only the primary activation of B cells but also the contribution of T cell help via cell-cell interactions and secreted cytokines to generate mature memory B cells and long-lived plasma cells capable of secreting high-titer specific antibodies. We designed our experiments not only to focus on the elements of this adaptive immune response but also to detect signs of underlying inflammation that could contribute to overall immune activation and thereby exacerbate adaptive responses. The immunophenotyping data were analyzed to identify any unique immunological characteristics associated with the HSAT response. In this hypothesis-generating pilot study, we aimed to: (1) provide insights into the basic mechanisms leading to immune sensitization against ERT, which could further guide the evolution of immunotherapy strategies; (2) to understand the impact of currently used ITI regimens on immune phenotypes in IOPD, and also (3) to ultimately guide the development of biomarkers and/or disease risk signatures for HSAT.

## 2 Materials and methods

### 2.1 Patients and inclusion criteria

Inclusion in the present study was based on; (a) a confirmed diagnosis of IOPD based on low GAA enzyme activity, two pathogenic GAA variants, and cardiomyopathy in the 1st year of life (2), (b) having received ERT with/without ITI, and (c) availability of sufficient blood sample and clinical data. Samples from the qualified patients were further classified into three groups based on (a) treatment history and (b) anti-rhGAA IgG antibody titer levels: (Group 1) IOPD patients who have received ERT monotherapy and have low anti-rhGAA IgG antibody titers (<12,800) without any immune modulation for >2 years, (Group 2) high-risk IOPD patients who have received the ITI protocol with rituximab, methotrexate ^+^/- IVIG in the ERT-naïve setting, received ERT for >2 years, and have low anti-rhGAA IgG antibody titers and B cell recovery at the time of sample collection, and (Group 3) IOPD patients who have developed HSAT.

### 2.2 Data collection

Clinical data including GAA variants, CRIM status, age at ERT initiation, time on ERT at the sample collection, and longitudinal anti-rhGAA IgG antibody titers were extracted from medical records. CRIM status was determined by western blot analysis in skin or blood at the Duke GSD/LSD Enzymology Laboratory and confirmed by GAA variants or was predicted based on known GAA variants as previously described (13). Anti-rhGAA IgG antibody titers were determined by Sanofi Genzyme or LabCorp by enzyme-linked immunosorbent assay and specificity was confirmed using radioimmunoprecipitation as previously described (5).

### 2.3 Isolation and storage of plasma and peripheral blood mononuclear cells (PBMCs)

Peripheral blood was obtained by venipuncture and collected in acid-citrate-dextrose tubes (BD Vacutainer, Franklin Lake, NJ). Plasma and PBMCs were separated by Ficoll (GE Healthcare, Uppsala, Sweden) density gradient centrifugation; the plasma layer was isolated first and aliquoted in 1mL increments and subsequently stored at −80°C. PBMCs were transferred to a new 50mL conical tube and washed, counted, and then re-suspended in a 90% FBS (Gemini, West Sacramento, CA) and 10% DMSO (Sigma-aldrich, St. Louis, MO) solution, and progressively cooled to −80°C in a CoolCell cell freezing container (BioCision, Larkspur, CA). The next day, the cells were stored in vapor phase liquid nitrogen for long-term storage.

### 2.4 Cellular analysis and flow cytometry

Cell preparation, staining, and analysis for flow cytometry were carried out by the Duke Immune Profiling Core (DIPC). PBMCs were thawed by washing twice with RPMI medium (R10) containing 10% FBS (Gemini Bio, Sacramento, CA) +1% penicillin, streptomycin, and L-glutamine (Invitrogen, Carlsbad, CA), and cell number and viability were calculated. A total of 10^6^ PBMCs were plated in 96 well round-bottom plates in R10. After centrifugation and removal of media, cells were surface stained with 50uL of an antibody cocktail mix consisting of titrated volumes of fluorescent antibodies for the innate and T cell panels. After fixing the cells with 1% paraformaldehyde (PFA), cells were acquired on a BD Symphony X50 flow cytometer (BD Biosciences, San Jose, CA). The antibodies listed in **Supplementary Table 1** from BioLegend (San Diego, CA) and BD Biosciences were used for flow cytometry.

### 2.5 Multiplex immunoassay

Plasma preparation and analysis for multiplex immunoassay were carried out by the DIPC. Patients’ plasma samples were used undiluted to measure the concentration of Fractalkine, GM-CSF, IFN-γ, IL−1β, IL-2, IL-4, IL-5, IL-6, IL-7, IL-8, IL-10, IL-12 (p70), IL-13, IL-17A, IL-21, IL-23, ITAC, MIP−1α, MIP−1β, MIP−3α, and TNF−α. Multiplex immunoassay was performed according to the manufacturer’s protocol (Millipore, HSTCMAG-28SK). Briefly, magnetic beads were diluted and added to a 96-well flat bottom plate. Plasma and serially diluted standards were added to wells in duplicate. The plate was incubated overnight and detection antibodies were added the next day. Lastly, Streptavidin-PE was added to each well prior to cytokine detection using a Magpix analyzer (Luminex, Austin, TX).

### 2.6 HSAT signature score derivation using principal components analysis (PCA)

Principal component analysis was performed on the flow cytometry and cytokine array data to generate PC1 loadings for HSAT signature parameters that were significantly different in both the comparisons between Group 2 vs. Group 3 and Group 1 + 2 vs. Group 3. Values were then z-score standardized across all samples within a parameter and multiplied by the PC1 loading values before summation to generate the HSAT signature score.

### 2.7 Statistical analysis

Following statistical analyses were performed after classifying the patient cohort into predetermined groups:

- Low antibody titers (LT; antibody titer <12,800) (Group 1 and 2) v/s High sustained antibody titer (HSAT; antibody titers ≥12,800) (Group 3); comparison between the group of patients which have high sustained antibodies (Group 3) and those who do not (Groups 1 + 2) to understand whether any of the flow and Luminex cytokine measures correlate with anti-rhGAA antibody status of the patients.
- Group 2 v/s Group 3. Both groups consist of patients at high-risk of developing anti-drug antibodies but with different treatment outcomes. The aim was to identify the immune characteristics in Group 3 compared to Group 2 which was successfully treated with ITI.
- Group 1 v/s Group 2; Both groups consist of “good responders” in terms of anti-rhGAA IgG antibodies as they maintained low anti-rhGAA IgG antibody titers. Group 2 was considered as high risk and as such received ITI in the ERT-naïve setting and were immune tolerant. The aim was to determine whether there were any immunological changes in Group 2 that might be associated with either their high-risk status and/or with administration of ITI or if looked similar in profile to Group 1 patients.

### 2.8 Study approval

All patients were enrolled in Duke institutional review board (IRB) approved study protocols Pro00001562 (Determination of Cross-Reactive Immunological Material [CRIM] Status and Longitudinal Follow-up of Individuals with Pompe disease), Pro00007612 (Genetic Disease Repository for Blood, Urine, and Tissue), and/or Pro00051144 (Identify alterations in phenotypically defined PBMC subpopulations and the possible generation of anti-Myozyme cellular reactivities in Pompe disease patients as a result of alglucosidase alfa). All patients were included in the study after provision of written informed consent by parents or a legally authorized representative.

## 3 Results

### 3.1 Description of patient groups

From pediatric IOPD patients that we follow at Duke, we identified a cohort of 40 patients with IOPD who met the criteria for one of the study groups (**Table 1**). A total of 40 PBMCs and 38 plasma deidentified samples were submitted to Duke Immune Profiling Core for flow cytometry and cytokine array analysis. Patients were divided into three groups based on antibody titers and ITI treatment status: (a) Group 1 (LT; n=20), long-term IOPD survivors who did not receive ITI and maintained low antibody titers, (b) Group 2 (ITI+LT; n=10), long-term high-risk IOPD survivors who received ITI with rituximab, methotrexate ^+^/- IVIG in the ERT-naïve setting and maintained low antibody titers, and (c) Group 3 (HSAT; n=10), IOPD patients who developed and maintained anti-rhGAA IgG antibody titers of ≥12,800 (including a mix of patients who received ITI and those who did not). Group 2 included CRIM-negative patients as well as CRIM-positive patients that were identified as high-risk based on family history or a combination of GAA variants that were previously seen in CRIM-positive patients with high antibody titers. The time that patients had been receiving ERT at the time of sample acquisition is closely correlated with age, and is included in **Table 1**, and a graph representing the close correlation (Spearman’s ρ = 0.9773, P<0.0001) is included in the **Supplementary Figures**.

**Table 1:** Patient characteristics.

Patient Code	Gender	*GAA* Variants		Age at sample (m)	Time on ERT at sample (m)	CRIM status	ITI in ERT-naïve setting
		Allele 1	Allele 2				
**1-01**	M	c.1650_1651dupG	IVS2 + 2_+5delTGGG	117.1	109.4	Negative	None
**1-02**	F	c.2297A>C	c.2297A>C	94.3	93.3	Positive	None
**1-03**	F	c.1650_1651dupG	IVS2 + 2_+5delTGGG	87.8	87.6	Negative	None
**1-04**	M	c.1933G>A	c.1933G>A	153.1	150.2	Positive	None
**1-05**	M	c.525delT	c.1642G>T, c.1880C>T	118.9	118.4	Positive	None
**1-06**	M	c.1564C>T	c.1221C>A, c.1281G>T, c.2296T>A	79.7	72.9	Positive	None
**1-07**	M	c.1933G>A	c.1933G>A	206.9	203.9	Positive	None
**1-08**	F	c.1802C>T	c.1099T>C	181.8	176.5	Positive	None
**1-09**	M	c.2297A>C	c.2297A>C	178.0	170.7	Positive	None
**1-10**	F	c.655G>A	c.655G>A	137.3	130.4	Positive	None
**1-11**	M	c.-32-13T>G	c.1447G>A	102.9	86.4	Positive	None
**1-12**	F	c.1978C>T	c.1477C>T, c.2221G>A	137.7	67.3	Positive	None
**1-13**	M	c.1327-2A>C	c.1327-2A>C	73.9	73.9	Positive	None
**1-14**	M	c.2560C>T	c.2560C>T	132.5	130.5	Negative	None
**1-15**	F	c.2481 + 102_2646 + 31del	c.670C>T	50.1	49.0	Positive	None
**1-16**	F	c.1293_1312del20	c.1716C>G	98.8	97.1	Positive	None
**1-17**	M	c.655G>A	c.2167_2179delinsTGCGACGTGG	35.2	31.7	Positive	None
**1-18**	F	c.2297A>C	c.2297A>C	124.7	120.8	Positive	None
**1-19**	M	c.1408_1410delAAC	c.925G>A	49.9	41.6	Positive	None
**1-20**	F	c.925G>A	c.1841C>A	96.9	94.9	Positive	None
**2-01**	F	c.2560C>T	c.2560C>T	60.3	59.0	Negative	RTX, MTX, IVIG
**2-02**	M	c.546 + 2_546+delTGGG	c.2501_2502delCA	40.0	38.4	Negative	RTX, MTX, IVIG
**2-03**	F	c.258dupC	c.2227C>T	55.8	52.7	Negative	RTX, MTX, IVIG
**2-04**	M	c.716delT	c.871C>T	39.5	33.1	Positive	RTX, MTX, IVIG
**2-05**	M	c.1827C>G	c.2662G>T	26.7	26.0	Negative	RTX, MTX, IVIG
**2-06**	F	c.1195-18_2190-20del	c.1195-18_2190-20del	26.0	21.6	Negative	RTX, MTX, IVIG
**2-07**	M	c.525delT	c.2560C>T	42.6	39.1	Negative	RTX, MTX, IVIG
**2-08**	M	c.525delT	c.2560C>T	25.3	23.9	Negative	RTX, MTX, IVIG
**2-09**	F	c.1841C>A	c2481 + 102_2646 + 31del	31.6	26.6	Positive	RTX, MTX, IVIG
**2-10**	M	c.2066_2070dup	c.2238G>A	18	17	Negative	RTX, MTX, IVIG
**3-01**	F	c.1195-18_2190-20del	c.1195-18_2190-20del	16.0	12.6	Negative	RTX, MTX, IVIG
**3-02**	F	c.670C>T	c.799_802delCTGATinsA	8.5	4.0	Positive	MTX
**3-03**	M	c.1754 + 2T>A	c.1822C>T	47.5	45.8	Negative	RTX, MTX, IVIG
**3-04**	F	c.2560C>T	c.2560C>T	113.0	112.6	Negative	None
**3-05**	M	c.925G>A	c.925G>A	14.4	9.5	Positive	None
**3-06**	F	c.1195-18_ 2190-20del	c.1195-18_ 2190-20del	81.4	75.3	Negative	RTX, MTX, IVIG
**3-07**	M	c.877G>A	c.716del	8.6	4.1	Positive	MTX
**3-08**	F	c.1418_1425delGCCAGCCG	c.2481G>C	21.5	15.1	Negative	RTX, MTX, IVIG
**3-09**	F	c.1210G>A	c.1210G>A	22.2	17.8	Positive	None
**3-10**	F	c.525delT	c.1979G>A	9.3	5.3	Positive	MTX

ITI, Immune tolerance induction; RTX, Rituximab; MTX, Methotrexate; IVIG, intravenous immunoglobulin; m, months; M. male; F, female; ERT, enzyme replacement therapy; CRIM, cross-reactive immunologic material.

### 3.2 High titer versus low titer group analysis

**Figure 1:**
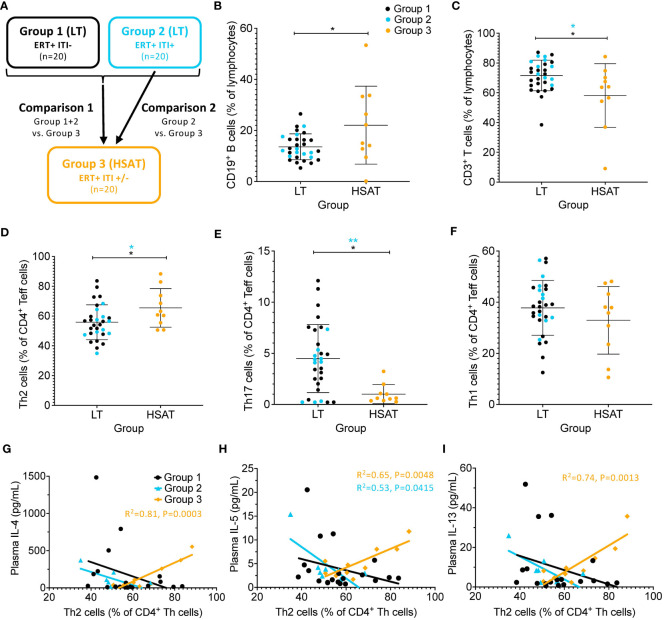
Skewing toward Type 2 immune profile in patients with HSAT. **(A)** Schema depicting the groups included in the study and comparisons between groups that are described in the text. Comparison of **(B)** CD19^+^CD3^-^ B cells and **(C)** CD3^+^CD19^-^ T cells (as a percentage of CD14- lymphocytes) between patients with HSAT at LT. **(D–F)** Comparison of Th1 (CXCR3^+^CCR6^-^), Th2 (CXCR3^-^CCR6^-^), and Th17 (CXCR3^-^CCR6^+^) cell-enriched populations between patients with HSAT at LT. Group 1 (LT: ERT+ ITI-) is identified in black, Group 2 (LT: ERT+ ITI+) in blue, and Group 3 (HSAT: ERT+ ITI+/-) in orange. *Age-adjusted P value (Padj) < 0.05, **Padj < 0.01. Color of asterix indicates results of testing between all LT (Group 1+2, black) or Group 2 (blue) and Group 3. **(G–I)** Correlation analysis (using Fisher’s exact test) between % Th2 cells (% of non-naïve CD4^+^ effector cells) and plasma concentrations of the Th2 cytokines (IL-4, IL-5, and IL-13). Regression lines for each group are superimposed in the corresponding color of group, as indicated in the legend. P-value and R2 for significant correlations are included on the graph in the color of the group in which the correlation exists.

A nested analysis approach was taken to interrogate the phenotypic immunological changes associated with the development of HSAT in patients with IOPD undergoing ERT (**Figure 1A**). In the first, broader comparison, all patients with HSAT (Group 3, n=10) were compared with all patients with LT (Group 1 combined with Group 2, n=30). This approach (LT vs. HSAT; Group 1 + 2 vs. Group 3) includes a greater number of patients (n=40) with a range of disease severity and effects, but also a significant range of ages, especially in Group 1 (**Tables 1**, **2**). With the goal of narrowing the analysis to optimally matched groups for comparison, we also carried out a focused sub-group analysis of only LT patients who have been treated with ITI (Group 2) compared to HSAT patients (Group 3). An additional feature of this sub-analysis is that it compares two groups that are more similar in age [Group 2 age 36.58 ^+^/- 13.72 months vs. Group 3 age 34.24 ^+^/- 35.87 months (mean ^+^/- SD)]. This is significant especially considering the unique features of immune phenotype and function in early childhood compared to that of older children and adults (16, 17). Patients in Groups 2 and 3 are all considered high-risk due to the CRIM-negative status and/or the expected severity of their identified variants. For each variable studied, regression analysis was carried out controlling for age at the time of sampling to identify parameters that were significantly different between groups.

**Table 2:** Summary of immunophenotyping parameters for individual groups.

	Group 1		Group 2		Group 3	
	Mean	SD	Mean	SD	Mean	SD
Age at sample (months)	112.87	45.50	36.58	13.72	34.24	35.87
Time of ERT at sample (months)	105.33	45.82	33.74	13.68	30.21	36.78
T Cell Parameters						
CD3^+^ T Cells	70.30	10.75	75.73	6.68	58.08	22.15
CD4^+^ T Cell Parameters						
% CD4^+^ T cells	63.48	5.90	69.87	7.91	67.24	15.22
CD4^+^ Naïve	75.67	7.56	83.78	3.10	83.52	11.36
CD4^+^ Central Memory	18.71	5.01	13.23	3.18	13.38	8.66
CD4^+^ Effector Memory	4.71	3.06	2.02	0.83	2.39	2.74
CD4^+^ TEMRA	1.10	0.76	1.07	0.62	0.79	0.68
CD4^+^ PD-1^+^	1.14	0.61	0.95	0.66	1.17	1.49
CD4^+^ TIGIT^+^	2.01	1.02	1.42	0.60	1.45	0.94
CD4^+^ CD38^+^	79.80	9.88	90.20	4.04	88.04	10.69
CD4^+^ CD38^+^HLA-DR^+^	0.82	0.55	1.02	0.88	2.38	4.77
CD4^+^ Treg (FoxP3^+^CD127^low^)	5.08	1.48	4.87	1.61	4.77	1.56
CD4^+^ Tfh	3.73	2.10	2.44	1.77	1.62	2.44
CD4^+^ Tfr	3.61	1.98	4.72	1.78	5.77	3.48
CD4^+^ Th1	36.22	11.27	40.91	9.22	32.91	13.19
CD4^+^ Th2	56.91	12.70	53.72	9.62	65.48	12.89
CD4^+^ Th17	4.96	3.64	3.54	2.45	1.01	0.93
CD8^+^ T Cell Parameters						
% CD8^+^ Cells	27.94	5.46	22.56	6.85	22.61	13.59
CD8^+^ Naïve	72.97	9.89	80.28	9.79	80.94	26.38
CD8^+^ Central Memory	2.21	0.84	2.15	0.92	2.54	1.74
CD8^+^ Effector Memory	13.32	6.19	7.42	4.55	12.31	25.42
CD8^+^ TEMRA	11.51	5.16	10.16	7.27	4.22	5.12
CD8^+^ PD-1^+^	1.83	0.91	1.42	0.68	1.18	1.40
CD8^+^ TIGIT^+^	6.32	3.67	3.87	1.92	3.44	2.78
B Cell Parameters						
CD19^+^ B cells	13.60	5.59	13.54	4.17	22.06	15.27
Transitional B cells	9.64	6.94	9.04	6.56	10.29	7.23
Naïve B cells	79.46	10.53	82.52	8.41	83.58	27.15
Unswitched memory B cells	1.86	0.95	1.36	0.71	0.57	0.44
Switched memory B cells	11.93	5.52	9.62	5.07	6.35	10.02
Plasmablasts	2.72	2.64	5.95	8.21	9.96	25.72
Monocyte Parameters						
CD14^+^ monocytes	71.11	12.00	64.60	17.19	70.52	13.97
Classical monocytes	93.02	6.46	95.54	2.26	92.93	5.31
Non-classical monocytes	5.95	6.31	3.78	2.34	5.79	4.84
Dendritic Cell Parameters						
Dendritic Cells	15.04	8.81	20.87	11.65	17.50	10.93
Myeloid Dendritic Cells (mDC)	39.85	10.41	39.34	12.89	29.47	10.67
Plasmacytoid Dendritic Cells (pDC)	10.33	7.97	9.73	6.35	8.26	8.36
NK Cells	6.75	4.27	4.48	3.26	3.88	3.51
CD16^+^CD56^+^ NK Cells	73.39	16.27	68.19	14.54	57.82	8.27
CD16^+^CD56^++^ NK Cells	2.44	2.54	3.46	2.10	3.71	3.29
CD16^-^CD56^++^ NK Cells	7.66	5.88	13.91	7.08	12.05	6.83
Cytokine Parameters						
CXCL11 (ITAC)	10.56	6.57	12.04	8.56	19.24	13.55
GM-CSF	24.62	20.87	42.43	23.68	19.96	9.35
CX3CL1 (Fractalkine)	44.15	12.38	61.41	19.14	60.20	12.39
IFN-y	11.21	4.97	17.10	16.35	14.00	4.41
IL-10	10.50	5.48	21.25	28.75	18.41	9.07
MIP-3α	10.61	6.64	18.36	9.00	17.34	7.66
IL-12 (p70)	1.92	0.92	2.27	2.15	2.41	1.25
IL-13	9.60	14.76	8.63	8.00	11.19	10.07
IL-17A	7.58	5.07	11.12	9.10	9.64	4.44
IL-1α	1.17	0.54	1.67	0.88	1.97	1.08
IL-2	1.52	1.40	2.36	2.16	1.82	0.63
IL-21	3.39	1.81	5.10	5.40	4.96	1.99
IL-4	183.47	364.99	111.53	122.14	154.62	181.22
IL-23	166.90	125.44	365.37	544.01	213.95	108.92
IL-5	3.97	4.93	4.65	4.41	5.25	3.17
IL-6	12.18	21.89	13.52	11.66	23.01	23.50
IL-7	7.89	3.70	10.22	4.04	9.22	2.36
IL-8	14.06	22.51	15.91	9.91	31.26	39.80
MIP-1α	18.43	34.70	18.05	19.57	16.68	11.14
MIP-1β	4.73	3.55	4.59	2.07	7.94	4.80
TNF- α	4.05	1.02	6.00	1.72	7.52	5.48

SD, standard deviation; m, months; ERT, enzyme replacement therapy.

#### 3.2.1 The circulating lymphocyte population is shifted toward increased B cell and decreased T cell frequencies in patients with HSAT

Phenotype analysis of lymphocyte subsets revealed that there was an increase in the percentage of CD19^+^ B cells (22.1 vs. 13.6% of lymphocytes, age-adjusted P value (Padj)=0.043) and a decrease in CD3^+^ T cells (58.1 vs. 72.1% of lymphocytes, Padj=0.018) in HSAT patients (Group 3) compared to LT patients (Group 1 + 2) (**Figures 1B, C**; gating strategy shown in **Supplementary Figure 1**). In the focused analysis comparing only Group 2 (ITI+LT) and Group 3 (HSAT), there was a persistent overall decrease in T cells (58.1 vs. 75.1% of lymphocytes, Padj=0.029), and an increase in B cells (22.1 vs. 13.5% of lymphocytes, Padj=0.11) in Group 3, though the difference in B cells did not reach significance.

#### 3.2.2 Patients with HSAT have increased circulating Th2 cells and decreased Th17 cells

Previous evidence in GAA-deficient mice treated with ERT with rhGAA demonstrated that anti-GAA IgG antibody responses are driven by immunodominant epitopes recognized by CD4^+^ T helper type-2 (Th2) cells (18). We carried out T helper (Th) subtype analysis in IOPD patients using well-established chemokine receptor expression patterns (19) to identify populations of CD4^+^ effector cells that are enriched for Th1 (Th1; CXCR3^+^CCR6^-^), Th2 (CXCR3^-^CCR6^-^), and Th17 (CXCR3^-^CCR6^+^) cells (**Figures 1D–F**, **Supplementary Figure 1**). There was an increase in mean Th2 (65.6 vs. 55.8% of CD4^+^; Padj=0.029) and a decrease in Th17 (1.0 vs. 4.5% of CD4^+^; Padj=0.012) cells in HSAT patients (Group 3) compared with LT (Group 1 + 2) patients (**Figures 1E, F**). There was not a significant difference in Th1 cells (32.9 vs. 37.8% of CD4^+^; Padj=0.17) (**Figure 1D**). In the focused sub-analysis, we again observed a significant increase in Th2 cells (65.5 vs. 53.7% of CD4^+^, Padj=0.032) and decrease in Th17 cells (1.0 vs. 3.5% of CD4^+^, Padj=0.009) in HSAT (Group 3) vs. ITI+LT (Group 2) patients, with no significant difference in Th1 cells (32.9 vs. 40.9% of CD4^+^, Padj= 0.14) (**Figures 1D–F**).

**Figure 2:**
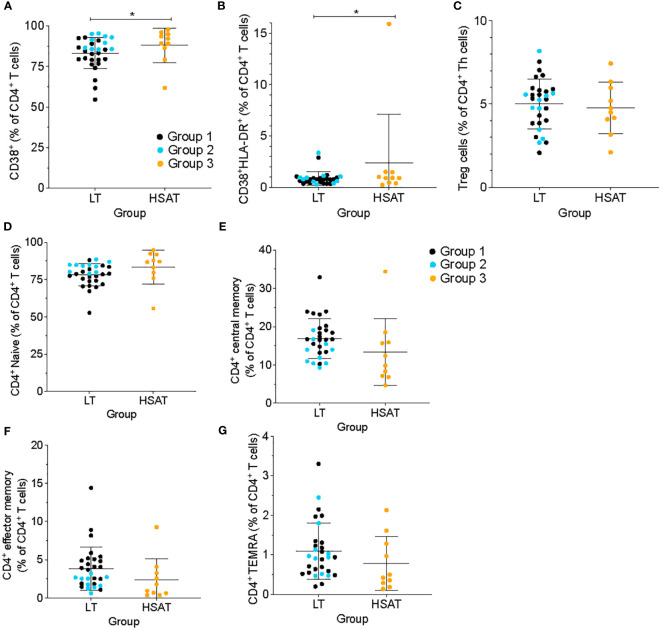
HSAT patients demonstrate evidence of T cell activation. Comparison of **(A)** CD4^+^CD38^+^ and **(B)** CD4^+^CD38^+^HLA-DR^+^ (as a percentage of CD4^+^ T cells), and **(C)** CD4^+^CD25^+^CD127^-^ T regulatory (Treg; as a percentage of CD4^+^CXCR5^-^ T cells) between HSAT and LT groups. **(D-G)** Comparison of maturation states of CD4^+^ T cells between HSAT and LT groups: Naïve (**D**, CD45RA^+^CCR7^+^), Central Memory (**E**, CD45RA^-^CCR7^+^), Effector/Effector Memory (**F**, CD45RA^-^CCR7^-^), and CD45RA^+^ Terminal Effector-Memory (**G**, TEMRA; CD45RA^+^CCR7^-^) cells. Group 1 (LT: ERT+ ITI-) is identified in black, Group 2 (LT: ERT+ ITI+) in blue, and Group 3 (HSAT: ERT+ ITI+/-) in orange. *Age-adjusted P value (Padj) < 0.05. Color of asterix indicates results of testing between all LT (Group 1 + 2, black) or Group 2 (blue) and Group 3.

In addition to Th1, Th2, and Th17 cells, we compared other CD4^+^ T cell subsets and found that there were no significant differences between HSAT patients and other groups in the levels of regulatory T cells (Treg; CD4^+^CD25^+^CD127^-^), T-follicular helper (Tfh; CD4^+^CXCR5^+^CD45RA^-^), or T-follicular regulatory (Tfr; CD4^+^CXCR5^+^CD45RA^-^CD25^+^CD127^-^) cells in circulation, though there was a clear trend toward decreased Tfh and increased Tfr in HSAT patients. (**Figure 2C**; **Supplementary Figures 2D, E**).

A shift toward increased Th2 cells in circulation suggests prior activation events leading to Th2 polarization, which is typically accompanied by an increase in Th2 cytokines at the local tissue level and may be reflected in the systemic circulation. This can be accompanied by a concomitant, reciprocal decrease in Th1 and/or Th17 cells and cytokines. In a comparison of mean Th1 (IFN-γ, IL-12), Th2 (IL-4, IL-5, IL-13), Th17 (IL-17, IL-21, IL-23), and immunoregulatory IL-10 cytokine concentrations in plasma, there were no significant differences detected in total plasma concentrations between HSAT and LT patients in either the larger or nested analysis (**Supplementary Figure 3**).

#### 3.2.3 Increased Th2 cells are correlated with higher levels of Th2 and proinflammatory cytokines in patients with HSAT

While there were no significant differences in the total plasma levels of Th1-, Th2-, or Th17-specific cytokines between treatment groups, we sought to understand if there were associations between cytokine levels and the skewed Th cell subsets observed in HSAT patients. Correlation analysis revealed a significant positive correlation between the frequency of Th2 cells and plasma concentrations of the Th2 cytokines IL-4 (R^2^= 0.81, P=0.0003), IL-5 (R^2^= 0.65, P=0.0048), and IL-13 (R^2^= 0.74, P=0.0013) that was only seen in HSAT patients (Group 3), but not other groups (**Figures 1G–I**). This was accompanied by a reciprocal negative correlation with Th1 cells (**Supplementary Figures 4A–C**). There were no correlations between Th1 or Th17 cells and canonical Th1 cytokines (IFN-γ, IL-12A) (**Supplementary Figures 4D, E**) or Th17 cytokines (IL-17A, IL-21, IL-23) (**Supplementary Figures 4G–I**), except for a positive correlation between IL-21 concentration and percent of Th17 cells in Group 3 (R^2^= 0.48, P=0.027) (**Supplementary Figure 4H**).

**Figure 3:**
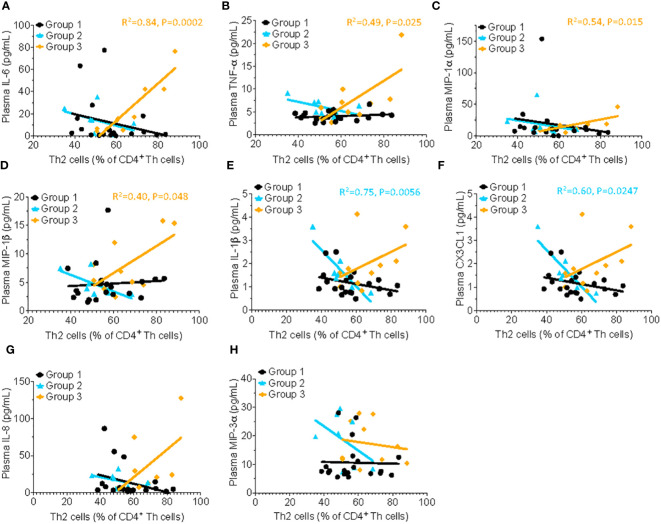
Th2 cells correlate with proinflammatory cytokine concentration in patients with HSAT. Correlation analysis (using Fisher’s exact test) between % Th2 cells (% of non-naïve Th effector cells) and plasma concentrations of the pro-inflammatory cytokines. Significant correlations were found only in Group 3 for IL-6 (**A**, R^2^= 0.84, P=0.0002), TNF-α (**B**, R^2^= 0.49, P=0.025), MIP-1α (**C**, R^2^= 0.54, P=0.015), and MIP-1β, (**D**, R^2^= 0.40, P=0.048), indicated by the orange regression line. Similar trends were observed for IL-1β **(E)**, CX3CL1 **(F)**, IL-8 **(G)** and MIP-3α **(H)** but failed to reach significance.

Several classical pro-inflammatory cytokines and chemokines, including IL-6 (R^2^= 0.84, P=0.0002), TNF-α (R^2^= 0.49, P=0.025), MIP-1α (R^2^= 0.54, P=0.015), and MIP-1β, (R^2^= 0.40, P=0.048) were positively correlated with the frequency of Th2 cells in HSAT patients (Group 3), but not in other groups (**Figures 3A–D**). There were significant inverse correlations between Th2 cells and the pro-inflammatory mediators IL-1β (R^2^= 0.75, P=0.0056) and CX3CL1 (also known as Fractalkine; R^2^= 0.60, P=0.0247) in Group 2 which appear to be reversed in HSAT patients (Group 3), though the positive correlation in HSAT patients fails to reach significance (**Figures 3E, F**). Similar trends were observed for IL-8 (CXCL8) and MIP-3a (**Figures 3G, H**), but failed to reach significance for either group.

#### 3.2.4 CD4^+^ T cells are more highly activated in patients with HSAT

We used cell surface markers of T cell activation (i.e., CD38 and HLA-DR) to compare the immune activation state between groups. CD4^+^ T cells from HSAT patients (Group 3) were more activated than LT patients (Group 1 + 2), as demonstrated by increased frequencies of CD38^+^ single positive (88.0 vs. 83.3% of CD4^+^ T cells, Padj=0.037) and CD38^+^HLA^-^DR^+^ double positive (2.28 vs. 0.88% CD4^+^ T cells, Padj=0.032) cells (**Figures 2A, B**). In the focused comparison of ITI+LT vs HSAT (Group 2 vs. 3), there was no significant difference. Increased activation can lead to the accumulation of memory populations and a consequent decrease in naive cells. We analyzed the maturation state of CD4^+^ T cells using standard markers of Naïve (CD45RA^+^CCR7^+^), Central Memory (CM; CD45RA^-^CCR7^+^), Effector/Effector Memory (EM; CD45RA^-^CCR7^-^), and CD45RA^+^ Terminal Effector-Memory (TEMRA; CD45RA^+^CCR7^-^) cells and found that there were no significant differences between groups (**Figures 2D–G**), though we observed a trend toward decreased circulating memory populations in the HSAT group.

#### 3.2.5 Circulating unswitched memory B cells are decreased in IOPD patients with HSAT

**Figure 4:**
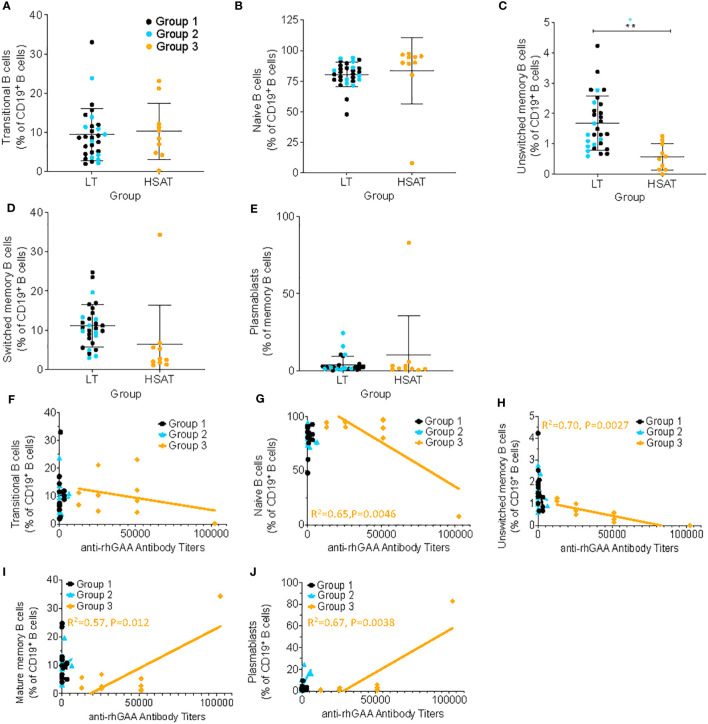
Deceased unswitched memory B cells and correlation of memory sub-populations with anti-rhGAA antibody concentration in HSAT patients. Comparison of percent of B cells (as percent of CD19^+^CD3^-^ B cells) in HSAT and LT patients along the continuum of B cell maturation, including: **(A)** transitional (CD19^+^CD20^+^CD38^hi^CD24^hi^), **(B)** naïve (CD19^+^IgD^+^CD27^-^), **(C)** unswitched memory (CD19^+^IgD^+^CD27^+^), **(D)** switched memory (CD19^+^IgD^-^CD27^+^), and **(E)** plasmablasts (CD19^+^CD27^+^IgD^-^CD20^-^CD38^++^). Group 1 (LT: ERT+ ITI-) is identified in black, Group 2 (LT: ERT+ ITI+) in blue, and Group 3 (HSAT: ERT+ ITI+/-) in orange. *Age-adjusted P value (Padj) < 0.01, ** (Padj) < 0.005. Color of asterix indicates results of testing between all LT (Group 1 + 2, black) or Group 2 (blue) and Group 3. **(F-J)** Correlation analysis (using Fisher’s exact test) between % B cell memory subgroup and anti-rhGAA antibody titers. Regression lines for each group are superimposed in the corresponding color of group, as indicated in the legend. P-value and R^2^ for significant correlations are included on the graph in the color of the group in which the correlation exists.

The hallmark clinical feature of Group 3 is the presence of high titer antigen-specific antibodies against rhGAA, which suggests an ongoing, mature, memory-type antibody response involving robust germinal center (GC) reactions in the lymphoid tissues (e.g., spleen and lymph nodes) and residence of long-lived plasma cells in the bone marrow. To determine whether the composition of circulating B cells reflects evidence of such a response, we analyzed the percentage of various maturational states of B cells in PBMCs, including transitional B cells (most nascent B cells in transit from bone marrow (BM) to periphery; CD19^+^CD20^+^CD38^++^CD24^++^), naïve B cells (antigen-specific but not activated; CD19^+^IgD^+^CD27^-^), unswitched memory B cells (previously activated in response to antigen but have not undergone a GC reaction and IgG heavy chain class-switching – argued to be the circulating equivalent of splenic marginal zone B cells; CD19^+^IgD^+^CD27^+^), switched memory (previously activated in response to antigen and matured by undergoing IgG heavy chain class-switching; CD19^+^IgD^-^CD27^+^), and plasmablasts (nascent antibody secreting cells typically in transit from the periphery to bone marrow; CD19^+^CD27^+^IgD^-^CD20^-^CD38^++^) (**Figures 4A–E**; gating scheme in **Supplementary Figure 1**) (20–23). We found that the frequency of circulating unswitched memory B cells in HSAT patients was around 1/3 of that which was observed in LT patients (0.57% vs. 1.68% of CD19^+^ B cells; Padj = 0.003) (**Figure 4C**). In the focused analysis comparing only LT patients who were treated with ITI (Group 2) vs. HSAT patients (Group 3), there was again a significant decrease in unswitched memory cells in the HSAT group (0.57% vs. 1.36% of CD19^+^ B cells; Padj=0.008) (**Figure 4C**). Titers of anti-rhGAA antibodies were negatively correlated with naïve (R^2^= 0.65 P=0.0046) and unswitched memory B cells (R^2^= 0.70 P=0.0027), and positively correlated with mature memory cells (R^2^= 0.57, P=0.012) and plasmablasts in Group 3 (R^2^= 0.67, P=0.0038) and (**Figures 4F–J**).

#### 3.2.6 Patients with HSAT have increased CXCL11 and decreased GM-CSF plasma concentration

**Figure 5:**
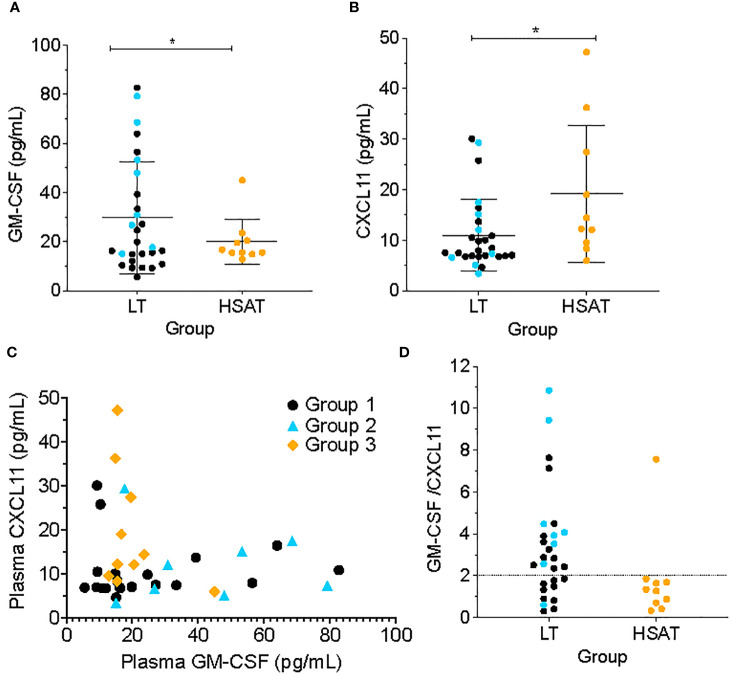
Low GM-CSF and high CXCL11 are characteristic of patients with HSAT. Comparison of plasma concentrations of **(A)** GM-CSF and **(B)** CXCL11 between HSAT and LT groups. *Age-adjusted P value (Padj) < 0.05. Color of asterix indicates results of testing between all LT (Group 1 + 2, black) or Group 2 (blue) and Group 3. **(C)** Correlation plot comparing GM-CSF and CXCL11 concentrations in Groups 1 (black), 2 (blue), and 3 (orange). **(D)**. Comparison of GM-CSF/CXCL11 ratio between LT (Groups 1 + 2) and HSAT (Group 3) patients. Dotted line indicates a reference value of GM-CSF/CXCL11 = 2.

Patients with HSAT (Group 3) had significantly higher average plasma concentrations of the chemokine CXCL11 (also known as ITAC; 19.24 vs. 10.98 pg/mL, Padj=0.037) and lower average plasma GM-CSF concentration (19.96 vs 29.71 pg/mL, Padj=0.038) compared with LT patient (Groups 1 + 2) (**Figures 5A, B**). In the focused subgroup comparison of ITI+LT (Group 2) vs. HSAT (Group 3) patients, only the decrease in GM-CSF concentrations observed in HSAT patients remained significant (19.95 vs. 42.42 pg/mL, Padj=0.018). There were trends toward increased levels of pro-inflammatory cytokines in Group 3 including IL-1β, IL-6, IL-8, MIP-1α, MIP-1β, MIP-3α, and TNF-α which did not reach significance in either the larger or subgroup analysis (**Supplementary Figure 3**).

Given that increased CXCL11 and decreased GM-CSF were observed in HSAT patients, we compared the concentrations of these cytokines across the entire cohort and found that there was a mutually inverse correlation between GM-CSF and CXCL11, such that all patients with high GM-CSF (>~20 pg/mL) also had low CXCL11 (<~20 pg/mL), while patients with high CXCL11 (> ~20 pg/ml) had low GM-CSF (<~20 pg/mL) (**Figure 5C**). Further, we confirmed that patients with HSAT tend to cluster according to their low GM-CSF and high CXCL11 levels (**Figure 5C**), though there is a significant overlap between groups. Thus, most patients with HSAT have a low ratio of GM-CSF/CXCL11 (below 2), but not all patients with a low GM-CSF/CXCL11 ratio have elevated Ab titers (**Figure 5D**).

#### 3.2.7 Derivation of an HSAT disease-associated signature score

**Figure 6:**
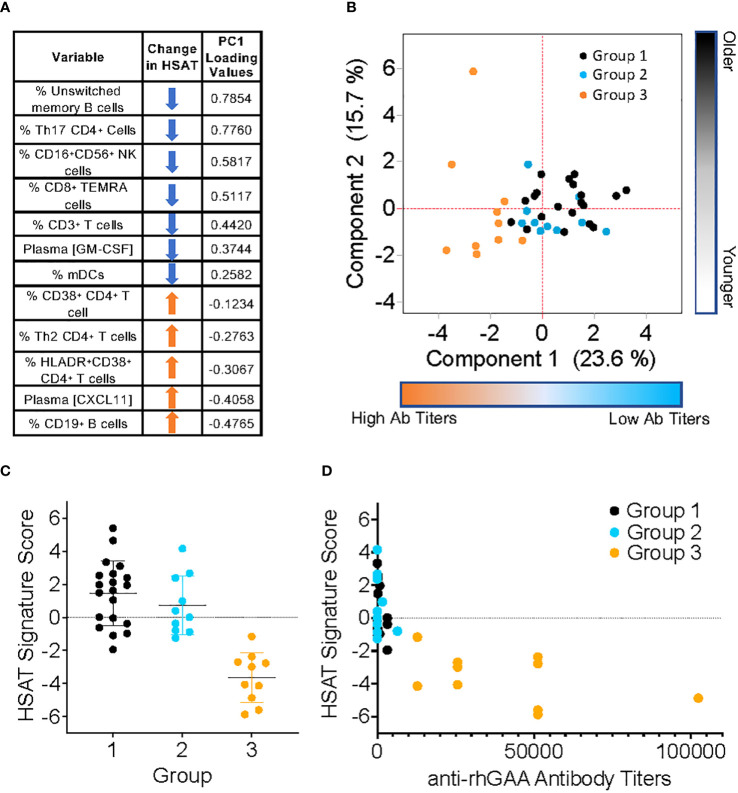
Derivation of PCA-weighted HSAT Signature Score. Principal Components Analysis (PCA) of the entire IOPD data set was carried out utilizing values of parameters that were significantly different between LT (Group 1+2) and HSAT (Group 3) patients. **(A)** Table of parameters included in PCA and their associated loading values in Principal Component (PC) 1. **(B)** Biaxial PCA plot demonstrating the distribution of Groups 1 (black), 2 (blue) and 3 (orange) along PC1 (Component 1) and PC2 (Component 2). **(C)** Comparison of HSAT Signature Score between Groups 1, 2, and 3. **(D)** Plot demonstrating correlation of HSAT signature score and anti-rhGAA antibody titers in Groups 1 (black), 2 (blue) and 3 (orange).

In addition to those described above, immunophenotyping revealed other significant differences in patients with HSAT compared to LT, including decreased CD11c^+^ myeloid dendritic cells (mDCs), CD16^+^CD56^+^ Natural Killer (NK) cells (**Table 3**, **Supplementary Figures 6A**, **7A**), and CD8^+^ TEMRA T cells (**Supplementary Figure 5D**). Given that multiple, diverse parameters were identified as being associated with development of HSAT in patients with IOPD, we set out to integrate these parameters into a potential immunophenotypic signature profile of patients who were in the HSAT group. We carried out principal component analysis (PCA) utilizing the measured values of parameters that were identified as significantly different in HSAT patients (listed in **Figure 6A**). Principal component 1 (PC1) accounted for 23.6% of variance in the data set, and patients from the cohort tend to segregate along PC1 according to their HSAT status, with Group 3 having more negative values in PC1, while LT groups centered more in the positive range of PC1 (**Figure 6B** and **Supplementary Figure 8B**). Patients that were more than 90 weeks old at the time of sampling were mostly in Group 1 and generally segregated according to positive values along PC2 (**Figure 6B**, **Supplementary Figure 8B**). Thus, patients tend to be distributed according to HSAT status along PC1. The PC1 loading values for each HSAT-signature immunophenotype parameter (e.g., GM-CSF concentration or % CD3^+^ T cells) were used to weight the z-standardized values of that parameter (**Figure 6A**, **Supplementary Figure 8A**), and the sum of the weighted values was taken to generate a standardized, PC1-weighted HSAT Signature Score, which was then calculated for everyone in the cohort. The HSAT signature scores were plotted by group (**Figure 6C**), and patients in Group 3 scored lower than patients in Groups 1 and 2, with minimal overlap between LT groups (Groups 1 and 2) and Group 3. Finally, antibody titers were compared to HSAT signature scores, demonstrating that elevated antibody titers correlated with low signature scores across the whole cohort (**Figure 6D**).

**Table 3:** Group comparisons of immunophenotyping parameters.

	Group 1 + 2 vs. Group 3 (LT vs. HSAT)				Group 2 vs. Group 3 (High-risk: LT vs. HSAT)				Group 1 vs. Group 2 (LT: No-ITI vs. ITI)			
	Mean		P value		Mean		P value		Mean		P value	
	Group 1 + 2	Group 3	Pnadj	Padj	Group 2	Group 3	Pnadj	Padj	Group 1	Group 2	Pnadj	Padj
T Cell Parameters												
CD3^+^ T Cells	72.11	58.08	0.01	0.02	75.73	58.08	0.03	0.03	70.30	75.73	0.16	0.17
CD4^+^ T Cell Parameters												
% CD4^+^ T cells	65.61	67.24	0.65	0.57	69.87	67.24	0.63	0.38	63.48	69.87	0.02	0.04
CD4^+^ Naïve	78.37	83.52	0.11	0.52	83.78	83.52	0.95	0.75	75.67	83.78	0.00	0.90
CD4^+^ Central Memory	16.88	13.38	0.13	0.58	13.23	13.38	0.96	0.81	18.71	13.23	0.00	0.99
CD4^+^ Effector Memory	3.81	2.39	0.17	0.29	2.02	2.39	0.69	0.36	4.71	2.02	0.01	0.59
CD4^+^ TEMRA	1.09	0.79	0.25	0.34	1.07	0.79	0.35	0.36	1.10	1.07	0.91	0.69
CD4^+^ PD-1^+^	1.08	1.17	0.79	0.40	0.95	1.17	0.68	0.44	1.14	0.95	0.44	0.35
CD4^+^ TIGIT^+^	1.81	1.45	0.30	0.94	1.42	1.45	0.92	0.83	2.01	1.42	0.10	0.40
CD4^+^ CD38^+^	83.27	88.04	0.20	0.04	90.20	88.04	0.56	0.09	79.80	90.20	0.00	0.25
CD4^+^ CD38^+^HLA DR^+^	0.88	2.38	0.09	0.03	1.02	2.38	0.39	0.16	0.82	1.02	0.44	0.76
CD4^+^ Treg (FoxP3^+^CD127^low^)	5.01	4.77	0.67	0.70	4.87	4.77	0.89	0.91	5.08	4.87	0.73	0.43
CD4^+^ Tfh	3.30	1.62	0.04	0.30	2.44	1.62	0.40	0.34	3.73	2.44	0.11	0.29
CD4^+^ Tfr	3.98	5.77	0.05	0.34	4.72	5.77	0.41	0.43	3.61	4.72	0.15	0.95
CD4^+^ Th1	37.78	32.91	0.25	0.17	40.91	32.91	0.13	0.14	36.22	40.91	0.26	0.91
CD4^+^ Th2	55.84	65.48	0.03	0.03	53.72	65.48	0.03	0.03	56.91	53.72	0.49	0.75
CD4^+^ Th17	4.49	1.01	0.00	0.01	3.54	1.01	0.01	0.01	4.96	3.54	0.27	0.34
CD8^+^ T Cell Parameters												
% CD8^+^ Cells	26.14	22.61	0.27	0.99	22.56	22.61	0.99	0.85	27.94	22.56	0.03	0.09
CD8^+^ Naïve	75.40	80.94	0.34	0.55	80.28	80.94	0.94	0.92	72.97	80.28	0.07	0.73
CD8^+^ Central Memory	2.19	2.54	0.39	0.31	2.15	2.54	0.53	0.55	2.21	2.15	0.85	0.41
CD8^+^ Effector Memory	11.35	12.31	0.85	0.10	7.42	12.31	0.56	0.32	13.32	7.42	0.01	0.94
CD8^+^ TEMRA	11.06	4.22	0.00	0.02	10.16	4.22	0.05	0.05	11.51	10.16	0.56	0.73
CD8^+^ PD-1^+^	1.69	1.18	0.17	0.55	1.42	1.18	0.63	0.62	1.83	1.42	0.21	0.20
CD8^+^ TIGIT^+^	5.50	3.44	0.09	0.90	3.87	3.44	0.69	0.74	6.32	3.87	0.06	0.79
B cell Parameters												
CD19^+^ B cells	13.58	22.06	0.01	0.04	13.54	22.06	0.11	0.11	13.60	13.54	0.98	0.88
Transitional B cells	9.44	10.29	0.74	0.74	9.04	10.29	0.69	0.73	9.64	9.04	0.82	0.18
Naïve B cells	80.48	83.58	0.59	0.74	82.52	83.58	0.91	0.96	79.46	82.52	0.43	0.74
Unswitched memory B cells	1.69	0.57	0.00	0.00	1.36	0.57	0.01	0.01	1.86	1.36	0.15	0.23
Switched memory B cells	11.16	6.35	0.06	0.43	9.62	6.35	0.37	0.22	11.93	9.62	0.28	0.84
Plasmablasts	3.80	9.96	0.21	0.10	5.95	9.96	0.64	0.38	2.72	5.95	0.12	0.23
Monocyte Parameters												
CD14^+^ monocytes	68.94	70.52	0.76	0.42	64.60	70.52	0.41	0.43	71.11	64.60	0.24	0.61
Classical monocytes	93.86	92.93	0.65	0.23	95.54	92.93	0.17	0.19	93.02	95.54	0.24	0.82
Non-classical monocytes	5.23	5.79	0.77	0.27	3.78	5.79	0.25	0.27	5.95	3.78	0.31	0.68
Dendritic Cell Parameters												
Dendritic Cells	16.98	17.50	0.89	0.58	20.87	17.50	0.51	0.54	15.04	20.87	0.14	0.60
Myeloid Dendritic Cells (mDC)	39.68	29.47	0.02	0.01	39.34	29.47	0.08	0.03	39.85	39.34	0.91	0.73
Plasmacytoid Dendritic Cells (pDC)	10.13	8.26	0.50	0.60	9.73	8.26	0.66	0.67	10.33	9.73	0.84	0.92
NK Cells	5.99	3.88	0.15	0.48	4.48	3.88	0.70	0.74	6.75	4.48	0.15	0.25
CD16^+^CD56^+^ NK Cells	71.66	57.82	0.01	0.03	68.19	57.82	0.07	0.08	73.39	68.19	0.40	0.20
CD16^+^CD56^++^ NK Cells	2.78	3.71	0.34	0.64	3.46	3.71	0.85	0.86	2.44	3.46	0.28	0.62
CD16^-^CD56^++^ NK Cells	9.75	12.05	0.36	0.89	13.91	12.05	0.56	0.55	7.66	13.91	0.02	0.05
Cytokine Parameters												
CXCL11 (ITAC)	10.98	19.24	0.02	0.04	12.04	19.24	0.21	0.19	10.56	12.04	0.62	0.96
GM-CSF	29.71	19.96	0.20	0.04	42.43	19.96	0.01	0.02	24.62	42.43	0.06	0.33
CX3CL1 (Fractalkine)	49.08	60.20	0.06	0.63	61.41	60.20	0.87	0.84	44.15	61.41	0.01	0.20
IFN-y	12.89	14.00	0.73	0.98	17.10	14.00	0.57	0.60	11.21	17.10	0.15	0.20
IL-10	13.58	18.41	0.38	0.53	21.25	18.41	0.77	0.81	10.50	21.25	0.11	0.13
MIP-3α	12.83	17.34	0.13	0.98	18.36	17.34	0.80	0.74	10.61	18.36	0.02	0.45
IL-12 (p70)	2.02	2.41	0.43	0.24	2.27	2.41	0.87	0.75	1.92	2.27	0.54	0.34
IL-13	9.32	11.19	0.68	0.49	8.63	11.19	0.57	0.58	9.60	8.63	0.86	0.54
IL-17A	8.59	9.64	0.64	0.99	11.12	9.64	0.66	0.68	7.58	11.12	0.20	0.36
IL-1β	1.31	1.97	0.03	0.14	1.67	1.97	0.54	0.56	1.17	1.67	0.07	0.09
IL-2	1.76	1.82	0.90	0.76	2.36	1.82	0.46	0.49	1.52	2.36	0.23	0.38
IL-21	3.88	4.96	0.33	0.50	5.10	4.96	0.94	0.98	3.39	5.10	0.21	0.29
IL-4	162.91	154.62	0.94	0.68	111.53	154.62	0.57	0.59	183.47	111.53	0.59	0.75
IL-23	223.60	213.95	0.92	0.76	365.37	213.95	0.40	0.42	166.90	365.37	0.13	0.14
IL-5	4.16	5.25	0.51	0.35	4.65	5.25	0.74	0.74	3.97	4.65	0.74	0.25
IL-6	12.58	23.01	0.18	0.22	13.52	23.01	0.31	0.34	12.18	13.52	0.87	0.47
IL-7	8.56	9.22	0.62	0.65	10.22	9.22	0.52	0.50	7.89	10.22	0.15	0.56
IL-8	14.59	31.26	0.09	0.16	15.91	31.26	0.31	0.32	14.06	15.91	0.83	0.36
MIP-1α	18.31	16.68	0.87	0.84	18.05	16.68	0.85	0.84	18.43	18.05	0.98	0.95
MIP-1β	4.69	7.94	0.02	0.09	4.59	7.94	0.09	0.10	4.73	4.59	0.92	0.29
TNF-α	4.61	7.52	0.01	0.10	6.00	7.52	0.46	0.49	4.05	6.00	0.00	0.01

LT, low titer; HSAT, high and sustained antibody titer; ITI, immune tolerance induction; Pnadj, non age-adjusted P value; Padj, age-adjusted P value.

### 3.3 Immunophenotypic analysis of LT groups

ITI with a short course of rituximab, methotrexate, and IVIG has become a standard of therapy for CRIM-negative IOPD patients and prevents development of deleterious immune responses to ERT. We wanted to determine whether treatment with these potent immunomodulatory therapies, though limited in their duration of exposure, results in long-term changes in immune phenotype in treated patients. We compared a group of ERT-treated patients with no exposure to ITI (Group 1; n=20) to patients on ERT who were also treated with ITI (Group 2; n=10) due to their CRIM-negative status or the anticipated risk associated with their known mutation. Of note, none of the patients included in this analysis developed HSAT. Given the difference in ages between Group 1 and Group 2, we controlled for age of the patient at the time the sample was taken using linear regression analysis.

There were few statistically significant changes noted between ITI+ and ITI- groups, but these included a higher percentage of CD16^-^CD56^++^ NK cells (13.91 vs. 7.6% of all CD56^+^, Padj=0.049) and CD4^+^ T cells (69.87 vs. 63.48% of CD3^+^, Padj=0.035) in patients treated with ITI, who also had higher levels of TNF-α (5.99 vs. 4.05 pg/mL, Padj=0.01)(**Supplementary Figures 9A–C**). The increase in TNF-α is modest but notable given that it is higher in patients who have received immunosuppressive therapy due to their increased risk of developing HSAT and may therefore reflect their underlying disease and demonstrate chronic, smoldering inflammation. This highlights an important consideration in interpreting the results of this comparison; the differences observed may not only reflect the use of ITI, but also the underlying immunological state associated with the factors which put these patients at increased risk for HSAT (i.e., CRIM-negative, high-risk mutations), and which lead to the administration of ITI.

## 4 Discussion

The negative impact of IgG anti-drug antibodies on the efficacy of therapeutic protein treatment for IOPD has been appreciated since the first clinical trials of rhGAA. Two decades later, however, we continue to have a limited understanding of cellular and cytokine mediators that govern immune responses during treatment with enzyme replacement. To counteract the anti-rhGAA IgG antibody response, various immunomodulation approaches have been employed with varying degrees of success, and our approach has been to use a combination of a short course of rituximab, methotrexate, and IVIG. Since both B and T cells participate in germinal center responses that result in sustained high-titer antibodies, both B cell-targeting (rituximab) and T cell targeting (methotrexate) agents were incorporated into the regimen. While we have been able to demonstrate normalization of CD19^+^ B cells to baseline levels after depletion with rituximab, very little else is known about the immunological state of patients with IOPD, especially those who received ITI and those who develop HSAT. To address this gap in current knowledge, we conducted a pilot study to establish an immune profile of the cellular subsets and secreted cytokines in a cohort of IOPD patients treated with ERT with or without ITI.

It is critical to realize that any data from immune phenotyping of PBMC in the circulation must be interpreted with the understanding that most immune cell interactions and reactions occur in tissues, especially secondary lymphoid tissues such as spleen and lymph node (LN), and that the cellular composition of blood may or may not reflect that of a relevant tissue. For example, high blood levels of a particular cell may correlate either positively or negatively with levels in tissue. Much as road traffic is lower during work hours when people are generally gathered at workplaces rather than in transit, tissue sequestration of a particular cell type into a site of active immune reaction may result in a relative decrease in the circulating frequency of that cell. With blood immune phenotyping, we capture a snapshot of cell populations in transit between compartments at one point in time. As such, an understanding of established relevant immunological paradigms must be cautiously employed when inferring mechanisms from circulating cell populations and cytokine levels. Another important distinction to make is between cell frequency, which we report here, and absolute cell counts. While the frequency of a cell conveys the fractional representation of that cell type within a parent population, it often does not correlate directly with the absolute cell count, which is the number of cells per volume of blood. These two measures can have different biological and diagnostic values but must not be confused or considered equivalent.

### 4.1 An immunophenotypic signature that correlates with the development of HSAT

CRIM status has been an important tool for stratifying risk in IOPD patients, but alone it is not adequate to identify the risk of HSAT, as a significant number of CRIM-positive patients will go on to develop HSAT. The overall goal of this work was to conduct comprehensive immune profiling of cytokines and cell subsets to identify unique immunological features in patients who develop HSAT. These features may potentially serve as biomarkers representing a disease ‘signature’, which may in the future be refined and tested to identify patients at risk for developing HSAT. To that end, we conducted statistical analysis to compare age-adjusted group mean values for each parameter between IOPD patients on ERT who developed HSAT (Group 3) and all patients who did not (Group 1 + 2). We further compared only LT patients who received ITI (Group 2) with HSAT patients (Group 3), finding that most of the identified differences were consistent between these analyses. Given that several parameters were identified as being significantly altered in HSAT patients, we sought to integrate these measurements to account for their contribution to development of HSAT. We included all significantly different parameters in PCA, revealing that patients were distributed along PC1 according to their antibody titers and therefore their HSAT status. Given the small number of parameters included and the natural degree of variability found in human phenotyping data, HSAT patients did not form a purely discrete cluster but were clearly skewed toward the more negative values of PC1. Loading values in PC1, which roughly represent the degree to which a particular parameter (e.g., % CD3^+^ T cells) contributes to the difference between HSAT and LT patients, were used to weight the measured values for each parameter and all weighted values were combined to derive a single numerical HSAT Signature Score. When patients in the cohort were scored according to this method, there was a clear separation between patients with HSAT and those without. While these data and analyses provide confirmatory evidence that the variables identified are associated with the presence of HSAT in this cohort, it is important to recognize that the interpretation of these results and their significance should be limited to this study and this cohort at this time. True evaluation of such a signature score requires a test for validation in a separate and independent cohort, but the results of the current pilot study could be used to rationally guide such endeavors.

In addition to providing the basis for a potential disease score measurement, the immunological parameters that were significantly different in patients with HSAT may also illuminate the pathophysiology underlying the development of HSAT. Keeping in mind the potential caveats mentioned above regarding the interpretation of cell frequencies in peripheral blood, we can learn a great deal about disease process by making careful interpretations and informed speculations regarding mechanisms of tissue-based reactions as reflected in circulating populations.

### 4.2 A shift toward type 2 immunity may support B cell maturation and production of HSAT

While antibodies can be generated without help from T cells (i.e. T-independent) or in a T cell-dependent manner that doesn’t involve maturation in a germinal center (GC), such antibody responses are typically short-lived and self-limited and lead to the production of lower titer and lower avidity antibody (16). Development of a highly specific, robust, and sustained antibody response such as HSAT against rhGAA involves the recruitment of B cells into GCs, where interactions with Tfh cells promote antibody affinity maturation, isotype class switching (i.e., from IgM and IgD to IgG, IgA, or IgE) (17, 18), and development of long-lived memory and antibody-secreting plasma cells. We observed a relative expansion of total B cells in the circulation of patients with HSAT that is accompanied by a shift in the circulating B cell population away from unswitched memory (CD27^+^IgD^+^) B cells. That this occurs in concert with high-titer specific IgG production suggests that the whole-body B cell compartment may be shifted toward a more mature type of memory and antibody-secretion response. While we did not detect a statistically significant difference in mature memory B or Tfh cells using robust testing methods, there is a clear visual decrease in the population of circulating mature memory B and Tfh cells in HSAT patients (**Figure 4** and **Supplementary Figure 2**). This may reflect recruitment and/or sequestration of Tfh cells out of the circulation into the secondary lymphoid tissues where they can participate in GC responses. This may also help to explain the observed HSAT-associated decrease in circulating unswitched memory cells since it could reflect a dominant shift toward GC-associated B cell reactions that are committed to generating mature, tissue-associated memory and antibody secreting cells (i.e., plasma cells). This is further supported by our observation that anti-rhGAA antibody titers are positively correlated with mature memory B cells and plasmablasts, and negatively correlated with naïve and unswitched memory B cells.

### 4.3 The makeup of the Th cell compartment in HSAT patients is significantly shifted toward Th2 cells, which may support the maturation of B cells into HSAT-producing cells

While Tfh cells play a key role in driving GC reactions that are essential for mature, robust, prolonged antibody responses, extrafollicular Th2 cells can also contribute to such antibody responses (16, 19). IL-4 and IL-13, which are secreted by Th2, Tfh, and several innate-type cells, are typically associated with atopic disease as well as immunity against extracellular parasites (e.g., helminths), but they also play an important broader role in humoral immunity. These cytokines not only drive B cell switching to IgG1 and IgE isotypes, but also act on B cells to support GC formation in type-2 immune responses, promote B cell proliferation, and protect them from apoptosis by modulating glycolytic metabolism (20, 21). Thus, IL-4/IL-13-deficient mice have decreased GC B cells during type-2 responses, and IL-4 from Th2 cells was sufficient to restore GC formation by acting directly on B cells (19). In the GAA−/− 129SVE mouse model of Pompe Disease, robust IgG1 antibody generation against human rhGAA is driven by immunodominant CD4^+^ Th2 cell epitopes, and these affected mice with high IgG antibody titers ultimately die from IgE-mediated anaphylaxis (22, 23). Our analysis demonstrated that the concentrations of type 2 cytokines (IL-4, IL-5, and IL-13) were significantly correlated with the increased percentage of Th2 cells in patients with HSAT. Since type 2 cytokines signal in a positive feedback loop to support ongoing Th2 cell differentiation and function, the correlation between type 2 cytokines and Th2 cells may reflect an expansion of cells in response to increased cytokine stimulation and/or increased secretion of cytokines by the expanded Th2 cell population. If activated Th2 cells contribute toward HSAT formation in humans, as they do in mice, an important question remains as to what mechanisms lead to their generation. A proposed summary model of how a dominant shift toward type 2 immunity in HSAT patients could feasibly contribute to the pathogenesis of HSAT is depicted in **Supplementary Figure 11**.

CRIM-negative patients are ideally set up to develop anti-rhGAA IgG antibodies. They lack endogenous expression of GAA, which normally drives the acquisition of immune tolerance through deletion of autoreactive T and B cell clones, and they undergo repeated administration of a specific antigenic stimulus (i.e., ERT with rhGAA). A quandary arises, therefore, in considering that patients in Group 2 who have the same immunological setup do not develop HSAT. The tolerizing regimens given to these patients due to their high-risk status allow them to maintain immune quiescence and avoid deleterious antibody responses through mechanisms of peripheral immune tolerance (e.g., influence of regulatory cells or tolerization of T or B cells through apoptosis or anergy). Conversely, some CRIM-positive patients do develop HSAT despite expressing endogenous GAA that should theoretically lead to natural immune tolerance. It is clear, therefore, that additional immunological and physiological factors are crucial for ‘tipping the scale’ from tolerance toward immunogenicity in HSAT patients. By examining parameters in our studies that define the immune profile of patients with HSAT (Group 3), we hope to gain insight into what factor(s) are responsible for this shift.

### 4.4 GM-CSF and CXCL11 form an immunological circuit that may contribute to the development of HSAT

Of all the cytokines and chemokines that were examined, only elevated plasma CXCL11 and decreased GM-CSF were significantly different in patients with HSAT compared to patients with LT. GM-CSF is a hematopoietic growth and differentiation factor for granulocytes and myeloid cells (e.g., monocytes and DCs), but it also plays an important part in myeloid cell activation, while CXCL11 is a chemokine originally described for its chemotactic properties, but which also has potent effects on immune cell signaling and differentiation (24–29). While high GM-CSF levels are strongly associated with disease in Multiple Sclerosis, Rheumatoid Arthritis, and other inflammatory diseases, decreased plasma GM-CSF is not commonly reported to be associated with disease states (25, 26, 28). There is, however, a clear association in Crohn’s Disease between decreased GM-CSF bioactivity and worsened disease severity (24, 28). Low GM-CSF in these patients has been linked to accelerated progression and increased gut permeability with resulting microbial translocation. In clinical trials, administration of recombinant GM-CSF to patients with moderate to severe Crohn’s was associated with increased rates of disease remission (28, 30). Furthermore, GM-CSF-null mice have impaired gut barrier function and are prone to intestinal damage in models of murine ileitis (31, 32), while administration of exogenous GM-CSF ameliorates colitis in these models and promotes healing of the mucosal epithelial barrier (33, 34), further highlighting the importance of normal GM-CSF function for maintaining intestinal barrier integrity. In addition to its role in mucosal healing and barrier integrity, GM-CSF may also play a role in maintaining a healthy intestinal microbiome (35).

We demonstrate that all patients in this IOPD cohort with elevated CXCL11 also have low GM-CSF, while all patients with high GM-CSF have low CXCL11. GM-CSF and CXCL11 are functionally related such that experimental antibody-mediated blockade of GM-CSF drives human monocytes to differentiate into CXCL11-producing immunomodulatory cells (24). CXCL11, in turn, was reported to inhibit T cell proliferation (24). The diminished levels of GM-CSF observed in patients with HSAT may therefore not only contribute to the increased levels of CXCL11 production in these patients but also to their decreased frequency of T cells due to the anti-proliferative influence of CXCL11 on these cells. CXCL11 both supports Th2 differentiation and inhibits Th17 differentiation *in vitro*, and administration of stabilized CXCL11-Ig fusion protein to mice resulted in decreased central nervous system Th1 and Th17 cells and increased Th2 and regulatory Tr1 cells in Experimental Autoimmune Encephalomyelitis (36, 37). CXCL11 also promotes susceptibility to cutaneous leishmaniasis in mice by promoting Th2 and inhibiting Th1 responses (38). These findings described in mice suggest that elevated levels of CXCL11 observed in HSAT patients may support the expansion of Th2 cells, promoting a dominant Type 2 milieu, and contributing to a paucity of Th17 cells.

Low GM-CSF levels may explain the decrease in mDC observed in patients with HSAT. While GM-CSF has typically been thought of as more important for monocytes and macrophage development and function, several studies clearly demonstrate a role for GM-CSF in the development of inflammation-induced mDC. The combined knockout of GM-CSF and Flt3-ligand severely reduced mDC production in mice, while genetic complementation by knock-in of human GM-CSF and Flt3-ligand resulted in enhanced mDC reconstitution and maturation in humanized mice (39–41).

Given that GM-CSF and CXCL11 can potentially influence multiple HSAT-associated parameters, we propose that these cytokines play a role in the pathogenesis of HSAT. In our theoretical model (**Supplementary Figure 10**), decreased plasma GM-CSF results in secretion of CXCL11 (24) which then acts on T cells to promote Th2 differentiation, inhibit Th17 differentiation, and decrease T cell proliferation, resulting in overall lower T cells with a Th2 predominance. The presence of increased T cell activation markers in HSAT patients suggests the influence of a broadly activating stimulus. We suggest that this chronic immune activation may be the result of microbial translocation due to impaired gut barrier integrity resulting from low GM-CSF concentrations. Decreased levels of Th17 cells, which we also observed in HSAT patients, could also contribute to microbial translocation since IL-17 is strongly associated with normal gut barrier integrity (42, 43). Both GM-CSF and IL-17A have been shown to promote colonic epithelial barrier integrity by enhancing expression and/or regulating protein trafficking of tight-junction proteins (44–46).

Gastrointestinal (GI) manifestations are common in patients with Pompe disease, including chronic diarrhea and/or constipation that has been assumed to be related to impaired GI smooth muscle motility, and the mouse model of Pompe disease (GAAKO 6neo/6neo) demonstrates accumulation of glycogen in smooth muscle throughout the GI tract (47). Given our findings, we suggest the possibility that these GI symptoms may in part be related to the immunological state of some IOPD patients, and specifically to low GM-CSF and/or Th17 levels. Low GM-CSF bioactivity due to anti-GM-CSF antibodies in Crohn’s Disease is associated with increased gut barrier permeability, microbial translocation, and innate immune activation (48), and it is now accepted that a large number of diverse pathological states are associated with altered gut barrier integrity (49). There is currently no direct evidence for increased gut permeability in IOPD, but it has not been specifically tested. Activation of innate immune cells in the gut by microbial products stimulating TLRs would result in the release of inflammatory mediators that then cause bystander immune activation of T and B cells. Several aspects of this model could be readily tested, especially it’s more speculative aspect: increased gut permeability and chronic immune activation due to microbial translocation. Given that it is unlikely to be explained by simple mendelian variation, the cause of low GM-CSF in HSAT patients remains unclear.

### 4.5 Evidence for chronic inflammation and immune activation in high-risk LT and HSAT+ patients

We found increased mean CD4^+^ T cell activation (HLADR^+^CD38^+^) in HSAT patients, compared to LT patients, suggesting the presence of a chronically activating stimulus. While levels of pro-inflammatory cytokine levels were not significantly different in HSAT patients, there were significant positive correlations between higher %Th2 cells and multiple pro-inflammatory cytokines, including IL-6, TNF-α, MIP-1α, and MIP-1β. There were also obvious but non-statistically significant correlations between Th2 cells and the pro-inflammatory cytokine IL-1β, CX3CL1, and IL-8 in HSAT patients. This may suggest that their increased risk for HSAT is associated with smoldering low-level inflammation due to either the primary impairment of glycogen handling and the resulting glycogen buildup, and/or low-level T and B cell activation in response to ongoing ERT exposure. Chronic gut leakiness would lead to chronic exposure to microbial products (e.g., LPS) that can cause innate immune activation. The resulting chronic inflammatory response, even at low levels, could play a role in secondary T and B cell activation, ultimately adding fuel to the fire of ongoing antibody responses. If chronic innate immune activation and inflammation are shown to contribute to HSAT responses (including breakthrough responses after ITI), it would suggest a role for therapeutic interventions that target the source of the inciting inflammatory triggers (e.g., gut leakiness resulting in LPS exposure) and/or the innate immune system’s inflammatory response to these triggers.

TNF-α was significantly elevated in high-risk LT patients in Group 2 who received ITI, compared to lower risk LT patients in Group 1 who did not. While this could be associated with the immunomodulatory therapy, they received prior to starting ERT, a more likely explanation is that some high-risk patients in Group 2 live with low-level chronic inflammation and immune activation despite receiving ITI. Thus, future studies may be warranted to determine if LT patients with elevated TNF-α are at higher risk for breakthrough development of HSAT. If so, therapies directed at blocking TNF-α and/or the upstream effects of IL-1β could be beneficial in preventing their conversion to HSAT production.

There is a notable absence of differences in CD4^+^ Treg cells between groups in this study. This was initially surprising, as it was hypothesized that increased production of regulatory immune cells (e.g., Tregs) could be responsible for suppressing anti-rhGAA responses in patients who had received ITI. The absence of differences in circulating Tregs does not, however, rule out this possibility. An expansion of rhGAA-specific Tregs at the time of ITI administration very likely would not be reflected in the frequency of overall circulating population, as such putative rhGAA-specific Tregs specific would carry out their suppressive function at the tissue level. Here, we enumerated the number of cells in circulation, but are unable to assess the frequency of cells in LNs and other tissues. We also were unable to assess the specificity or suppressive activity of Tregs, which are both crucial for understanding their functionality.

There are several important caveats associated with this study, including the relatively low number of patients. However, given the rarity of patients with IOPD and of HSAT patients within that group, this is the largest published series of IOPD patients to undergo multiparameter immunophenotyping. Even so, interpretations must be made with caution when considering the larger disease population. This is particularly true in the case of the PC1-weighted HSAT Signature Score we derived to integrate several HSAT-associated parameters. While the score appropriately stratifies the patients from which it is derived according to their antibody titers, validation requires studying these parameters and the HSAT Signature Score in an independent cohort. The available data on anti-rhGAA IgG antibody titers only detailed total antibodies and was unable to distinguish between neutralizing vs. binding (non-neutralizing) antibodies. In addition, prior data supports that the patients who develop high total IgG titers, even those with no abnormality noted in uptake or binding, showed suboptimal clinical response to ERT (5, 50). Additionally, the impact of anti-rhGAA IgG antibodies in patients with LOPD is not completely clear in contrast to the well-documented evidence on the deleterious effect in IOPD patients. As such it will be important to see if the findings of this study are also noted in a well-phenotyped cohort of LOPD patients.

A technical caveat regarding flow cytometry analysis is that we employed a widely used method for measuring the frequency of Th2 and Th17 cells via cell surface chemokine receptors. It must be recognized, however, that the definitive test to identify these cells is intracellular flow cytometry for canonical transcription factors and/or cytokines, which was not feasible for this pilot study but should be carried out in further confirmatory studies. We report a reciprocal relationship between Th2 cytokines (IL-4, IL-5, IL-13) and Th1 vs. Th2 cells. While it is well-recognized that Th1 and Th2 cells exert a reciprocal negative regulation on each other’s development through cytokine signals, it must also be considered that the reciprocity of these correlations also likely arises from the gating scheme which parses Th1 and Th2 cells based on CXCR3 positivity, and therefore does reveal an overall literal shift in the effector population toward Th2 cells based on surface phenotype. Finally, in our theoretical model, we propose a relationship between CXCL11 and T cell proliferation, enhanced Th2 differentiation, and impaired Th17 differentiation, but we did not see direct correlations between these measurements. This may be explained by the knowledge that most cytokines act most potently at the microscopic scale of cell-cell interactions, and plasma levels of cytokines may not always reflect these paracrine interactions. This caution applies when considering the importance of both statistically significant and non-significant associations.

### 4.6 HSAT immunophenotyping can inform future diagnostic and therapeutic approaches

With the implementation of ERT and ITI, CRIM-negative patients with IOPD are living longer than previously with improved quality of life, but are faced with a new set of challenges that potentially limit the success of their treatment, inducing development of HSAT. Understanding the immunobiology in the new emerging phenotype of long-term CRIM-negative IOPD survivors could help identify the factors that may lead to their clinical decline. Among the phenotypic traits studied, signs of chronic inflammation and immune activation are emerging as likely candidates underlying such progressive disease. There are potential implications that may be drawn from the findings in this study which may impact therapeutic strategies for mitigation of HSAT. Given that both T and B cells seem to be significantly affected in patients that develop HSAT, the addition of intensified T-cell targeted therapies (e.g., cyclosporine, sirolimus, tacrolimus, or mycophenolate mofetil) in addition to B-cell depletion with rituximab could more potently prevent the initial sensitization of T cells that go on to contribute to mature B memory cell and plasma cell responses. Given the vulnerable state of IOPD patients, the use of such agents would require careful consideration and may first be best evaluated in the rare patients who develop breakthrough HSAT after an initial round of ITI or in adults with Pompe disease. Furthermore, multiple lines of evidence presented here suggest a role for innate immune activation in driving the pathogenesis of HSAT. Biologic agents that block inflammatory cytokines and/or cytokine receptors (e.g., against IL-1β or TNF-α) may therefore have a therapeutic role in preventing or treating it. Recombinant preparations of GM-CSF have been widely used to support myeloid reconstitution after hematopoietic stem cell transplantation, to treat neutropenia during chemotherapy, or to ameliorate radiation-induced myelosuppression (51, 52). If low GM-CSF is found to play a likely role in the pathogenesis of HSAT, these off-the-shelf medicines could therefore be further studied and, if successful, incorporated into clinical trials.

## Data availability statement

The original contributions presented in the study are included in the article/**Supplementary Material**. Further inquiries can be directed to the corresponding authors.

## Ethics statement

The studies involving humans were approved by Duke University Health System Institutional Review Board. The studies were conducted in accordance with the local legislation and institutional requirements. Written informed consent for participation in this study was provided by the participants’ legal guardians/next of kin.

## Author contributions

AD: Conceptualization, Funding acquisition, Investigation, Methodology, Project administration, Resources, Supervision, Writing – original draft, Writing – review & editing. PS: Data curation, Formal analysis, Methodology, Validation, Writing – original draft, Writing – review & editing. JY: Conceptualization, Formal analysis, Investigation, Methodology, Resources, Writing – review & editing. AR: Formal analysis, Writing – original draft, Writing – review & editing. TB: Data curation, Formal analysis, Investigation, Visualization, Writing – original draft, Writing – review & editing. PK: Conceptualization, Funding acquisition, Investigation, Methodology, Supervision, Writing – original draft, Writing – review & editing.
