# Supplementary material for: Immunophenotype associated with high sustained antibody titers against enzyme replacement therapy in infantile-onset Pompe disease
Source: Front Immunol. 2024 Jan 4;14:1301912. doi: 10.3389/fimmu.2023.1301912 (PMC10798041; doi:10.3389/fimmu.2023.1301912)
Supplement: Supplementary file 1 [file DataSheet_1.pdf]

Supplemental Material

Immunophenotype associated with high sustained antibody titers against enzyme replacement treatment in Infantile Pompe Disease

Ankit K. Desai, P. Brian Smith, John S. Yi, Amy S. Rosenberg, Trevor D. Burt,* Priya S. Kishnani,*

Supplemental Figure Legends:

Supplemental Figure 1: Representative flow cytometry plots and gating strategies. A. Progressive gating strategy of PBMCs to identify T cell subsets, including: sub-gating of $CD4^+$ and $CD8^+$ T cells to identify $CCR7^+CD45RA^+$ Naïve, $CCR7^+CD45RA^-$ central memory (CM), $CCR7^-CD45RA^-$ effector memory (EM), $CCR7^-CD45RA^+$ terminal effector memory (TEMRA) cells; sub-gating of $CD4^+$ cells to identify $CD38^{+/hi}$ and activated $CD38^{+/hi}HLA\text{-}DR^+$ cells, Th subsets (Th1 enriched $CCR6^-CXCR3^+$ cells, Th2 enriched $CCR6^-CXCR3^-$ cells, and Th17 enriched $CCR6^+CXCR3^-$ cells), $CD45RA^-CXCR5^+$ T follicular helper (Tfh) cells, $CD45RA^-CXCR5^+CD25^+CD127^-$ T follicular regulatory (Tfr), and $CD25^+CD127^-$ T regulatory (Treg) cells. (B) Progressive gating strategy of PBMCs to identify B cell, NK cell, and myeloid cell subsets, including: subgating of $HLA\text{-}DR^+CD14^-$dendritic cells (DCs) to identify DC cell subsets ($CD11c^+CD123^-$ myeloid dendritic cells (mDCs) and $HLA\text{-}DR^+CD14^-CD11c^-CD123^+$ plasmacytoid dendritic cells (pDCs)); subgating of $HLA\text{-}DR^+CD14^+$ monocytes subsets to identify monocyte subsets ($HLA\text{-}DR^+CD14^+CD14^+CD16^-$ classical monocytes, and $HLA\text{-}DR^+CD14^+CD14^+CD16^+$ non-classical monocytes); subgating of $CD56^+$ NK cells to identify NK cell subsets ($CD16^+CD56^+$, $CD16^+CD56^{++}$, and $CD16^-CD56^{++}$ NK cells); and sub-gating of $CD19^+$ B cells to identify $CD19^+IgD^+CD27^-$ naïve, $CD19^+IgD^+CD27^+$ unswitched memory, $CD19^+IgD^-CD27^+$mature memory, and $CD19^+CD20^+CD38^{++}CD24^{++}$ transitional B cells as well as $CD19^+CD27^+IgD^-CD20^-CD38^{++}$ plasmablasts.

Supplemental Figure 2: Comparison of T cell subsets. Comparison of (A) $CD4^+$ T cells and (B) $CD8^+$ T cells as a percentage of $CD3^+$ T cells between patients with HSAT and LT. (C) Ratio of $CD4^+$ and $CD8^+$ T cells. (D) $CD4^+CD45RA^-CXCR5^+$ T helper cells (Tfh; as a percentage of $CD4^+$ T cells) and (E) $CD4^+CD45RA^-CXCR5^+CD25^+CD127^-$ T follicular regulatory (Tfr; as a percentage of Tfh cells) cells between patients with HSAT and LT. Group 1 (LT: ERT+ ITI-) is identified in black, Group 2 (LT: ERT+ ITI+) in blue, and Group 3 (HSAT: ERT+ ITI+/-) in orange.

Supplemental Figure 3: Comparison of cytokines and chemokines between IOPD groups. Comparison between Group 1 (black dots and lines), Group 2 (blue dots and lines), and Group 3 (orange dots and lines) for measured plasma values of: (A) IFN-γ, (B) IL-12 (p70), (C) IL-10, (D) IL-4, (E) IL-5, (F) IL-13, (G) IL-17A, (H) IL-21, (I) IL-23, (J) IL-8, (K) IL-1β, (L) TNF-a, (M) MIP-1α, (N) MIP-1β, (O) MIP-3 α, (P) CX3CL1, (Q) IL-2, and (R) IL-7.

Supplemental Figure 4: Correlations between Th1 and Th17 cell percentages and plasma cytokine levels.
(A-F) Correlation analysis (using Fisher's exact test) between % Th1 cells (% of non-naïve $CD4^+$ effector cells) and plasma concentrations of the Th2 and Th1 cytokines (A) IL-4, (B) IL-5, (C) IL-13, (D) IL-12, and (E) IFN-γ, as well as (F) IL-8. (G-I) Correlation analysis (using Fisher's exact test) between % Th17 cells (% of non-naïve $CD4^+$ effector cells) and plasma concentrations of the Th17 cytokines (G) IL-17A, (H) IL21, (I) IL-23. Regression lines for each group are superimposed in the corresponding color of group, as indicated in the legend. P-value and R^2 for significant correlations are included on the graph in the color of the group in which the correlation exists.

Supplemental Figure 5: Maturation stages of $CD8^+$ cells. Comparison of maturation states of $CD8^+$ T cells between HSAT and LT groups: (A) $CD45RA^+CCR7^+$ naïve, (B) CD45RA-$CCR7^+$ central memory, (C) CD45RA-$CCR7^-$ effector memory, and (D) $CD45RA^+CCR7^-$ terminal effector memory (TEMRA) cells. Group 1 (LT: ERT+ ITI-) is identified in black, Group 2 (LT: ERT+ ITI+) in blue, and Group 3 (HSAT: ERT+ ITI+/-) in orange. *Age-adjusted P value (Padj) < 0.05. Color of asterisk indicates results of testing between Group 1 (black) or Group 2 (blue) and Group 3 (orange).

Supplemental Figure 6: Comparison of myeloid cell subsets. (A,B) Comparison of DC subsets as a percentage of HLA-DR^+CD14^- myeloid cells, including: (A) HLA-$DR^+CD14^-CD11c^+CD123^-$ myeloid dendritic cells (mDCs) and (B) HLA-$DR^+CD14^-CD11c^-CD123^+$ plasmacytoid dendritic cells (pDCs). (C,D) Comparison of HLA-DR^+CD14^+ monocytes subsets, including: (C) HLA-$DR^+CD14^+CD14^+CD16^-$ classical monocytes, and (D) HLA-$DR^+CD14^+CD14^+CD16^+$ non-classical monocytes. *Age-adjusted P value (Padj) < 0.05. Color of asterisk indicates results of testing between Group 1 (black) or Group 2 (blue) and Group 3 (orange).

Supplemental Figure 7: Comparison of natural killer (NK) cell subsets. Comparison of NK cell subsets as a percentage of $CD56^+$ NK cells, including: (A) $CD16^+CD56^+$, (B) $CD16^+CD56^{++}$, and (C) $CD16^-CD56^{++}$,. *Age-adjusted P value (Padj) < 0.05. Color of asterisk indicates results of testing between Group 1 (black) or Group 2 (blue) and Group 3 (orange).

Supplemental Figure 8. Immunophenotypic parameters included in derivation of PCA-weighted HSAT Signature Score. Principal Components Analysis (PCA) of the entire IOPD data set was carried out utilizing values of parameters that were significantly different between LT (Group 1+2) and HSAT (Group 3) patients. (A) Histogram representing PC1 loading values of parameters included in PCA. (B) Biaxial PCA plot demonstrating the contribution of immunophenotype parameters, arrows represent interaction of PC1 and PC2 eigenvectors. (C) Heatmap showing measured values of all parameters that were significantly different between HSAT and LT groups. Colors represent relative, Z score, log-normalized expression across each parameter. Top rows (HSAT status and study group) included for reference, not included in clustering.

Supplemental Figure 9. Immunophenotypic analysis of LT groups. Comparison of parameters that were significantly different between Group 1 (LT: ERT+ITI-) and Group 2 (HSAT: ERT+ITI+), including: (A) Plasma TNF-a concentration, (B) $CD16^-CCR7^{++}$ NK cells, and (C) $CD4^+$ T cells. *Age-adjusted P value (Padj) < 0.05. Color of asterisk indicates results of testing between Group 1 (black) or Group 2 (blue) and Group 3 (orange).

Supplemental Figure 10
Theoretical model depicting the role of decreased [GM-CSF] and increased [CXC11] in driving the pathogenesis of HSAT. In IOPD patients with HSAT, decreased GM-CSF results in uninhibited production of CXCL-11, which has multiple effect on T cells, including: decreased proliferation, resulting in lower percentage of all T cells, enhancement of Th2 cell differentiation and inhibition of Th17 cell differentiation. Low GM-CSF availability and decreased IL-17A

from Th17 cells may result in impaired intestinal barrier integrity, leading to translocation of microbial products. Gut-resident innate immune cells are stimulated and activated by microbial products result to produce inflammatory mediators that activate T and B cells. Activated, IL-4-producing Th2 cells then may go on to drive HSAT responses (see Figure 11). Orange boxes and arrows represent actual observed changes and correlations, while black arrows and non-highlighted text represented theoretical changes and mechanisms based on established immunological pathways and interactions observed in other settings.

Supplemental Figure 11. Model of rhGAA-specific (α-rhGAA) antibody responses in patients with Infantile Pompe Disease (IOPD) treated with rhGAA. Theoretical scenarios explain the disparate antibody responses in patients with low titers (LT) and high sustained antibody titers (HSAT) against rhGAA are explored based on the current understanding of how B cell memory and antibody secreting cells (e.g., plasmablasts and plasma cells) are generated in response to antigens challenge in humans [1-5].

A. In non-HSAT (LT) IOPD patients, administration of rhGAA allows for (1) stimulation of naïve rhGAA-specific (α-rhGAA) B cells resulting of priming and early activation, including differentiation into IgD^+CD27^+ unswitched memory B cells (*see below), (2) stimulation of a α-rhGAA T naïve $CD4^+$ T cells in the context of signals (e.g. cytokines) that allow for (3) differentiation into α-rhGAA T follicular helper (Tfh) cells. (4) In the absence of sufficient α-rhGAA T/B cells and/or appropriate signals/stimuli, geminal centers (GCs) fail to form and robust antibody responses against rhGAA are not generated. (5) Low numbers of short lived a-rhGAA plasma cells and IgD^+CD27^+ unswitched memory B cells (*see below) may generated by extra-follicular differentiation pathways, [3, 6] resulting in low titers of α-rhGAA Abs.

B. In patients with residual endogenous rhGAA expression (i.e. CRIM+) and/or in the setting of immune tolerance induction (ITI), (7) tolerogenic cells (Tregs, Bregs, Tfr) may inhibit T and B cell activation, thereby preventing antibody responses.

C. In IOPD patients with HSAT, rhGAA provides chronic stimulation, while factors such immune activation and (8) IL-4 produced by an explanded population of Th2 cells promote GC formation. In the GC, α-rhGAA B cells (9) proliferate robustly, undergo selection, somatic hypermutation, and class-switch recombination resulting in (10) production of mature memory α-rhGAA B cells and antibody secreting cells (plasmablasts and plasma cells). (11) Long lived plasma cells migrate to bone marrow where they secrete α-rhGAA antibodies, resulting in high, sustained antibody titers. (12) Mature memory α-rhGAA B cells may recirculate to LNs where they can be rapidly and efficiently recruited into GC reactions to amplify the cycle of memory and plasma cell generation. In this scenario, B cells may be shunted away from T-independent and immature memory phenotypes, resulting decreased unswitched IgD+CD27+ memory cells

* The origins and functions of unswitched IgD+CD27+ memory cells (largely comprised of IgD+IgM+CD27+ cells) in humans is widely debated, and almost certainly represent a non-homogenous population. They have been postulated to represent the circulating equivalent of splenic marginal zone B cells, memory B cells which arose from T-independent stimulation in extrafollicular reactions, and/or cells that arise from early stages of GC reactions[5, 6]. They are transcriptionally similar to mature, class-switched memory cells, seem to have a predisposition to enter into GC reactions upon re-stimulation[5]. Thus, repeated exposure to rhGAA might also support the exhaustion of this cell population as they too enter into GC reactions in ultimately become mature memory B cells.

Supplemental Figure 12. Correlation between time on ERT at sample collection and age at sample collection. Comparison of the age at which the sample was drawn for this study and the time that the patient who donated the sample had been treated with ERT at the time of sampling. Time is expressed in months. Spearman correlation analysis was carried out and values for Spearman's r and P value are included in the figure. A line of simple linear regression is included for visualization.

1. Crotty, S., *T Follicular Helper Cell Biology: A Decade of Discovery and Diseases.* Immunity, 2019. **50**(5): p. 1132-1148.
2. Nutt, S.L., et al., *The generation of antibody-secreting plasma cells.* Nat Rev Immunol, 2015. **15**(3): p. 160-71.
3. MacLennan, I.C., et al., *Extrafollicular antibody responses.* Immunol Rev, 2003. **194**: p. 8-18.
4. Cyster, J.G. and C.D.C. Allen, *B Cell Responses: Cell Interaction Dynamics and Decisions.* Cell, 2019. **177**(3): p. 524-540.
5. Kibler, A., M. Seifert, and B. Budeus, *Age-related changes of the human splenic marginal zone B cell compartment.* Immunol Lett, 2023. **256-257**: p. 59-65.
6. Sanz, I., et al., *Challenges and Opportunities for Consistent Classification of Human B Cell and Plasma Cell Populations.* Front Immunol, 2019. **10**: p. 2458.

Supplemental Figure 1A

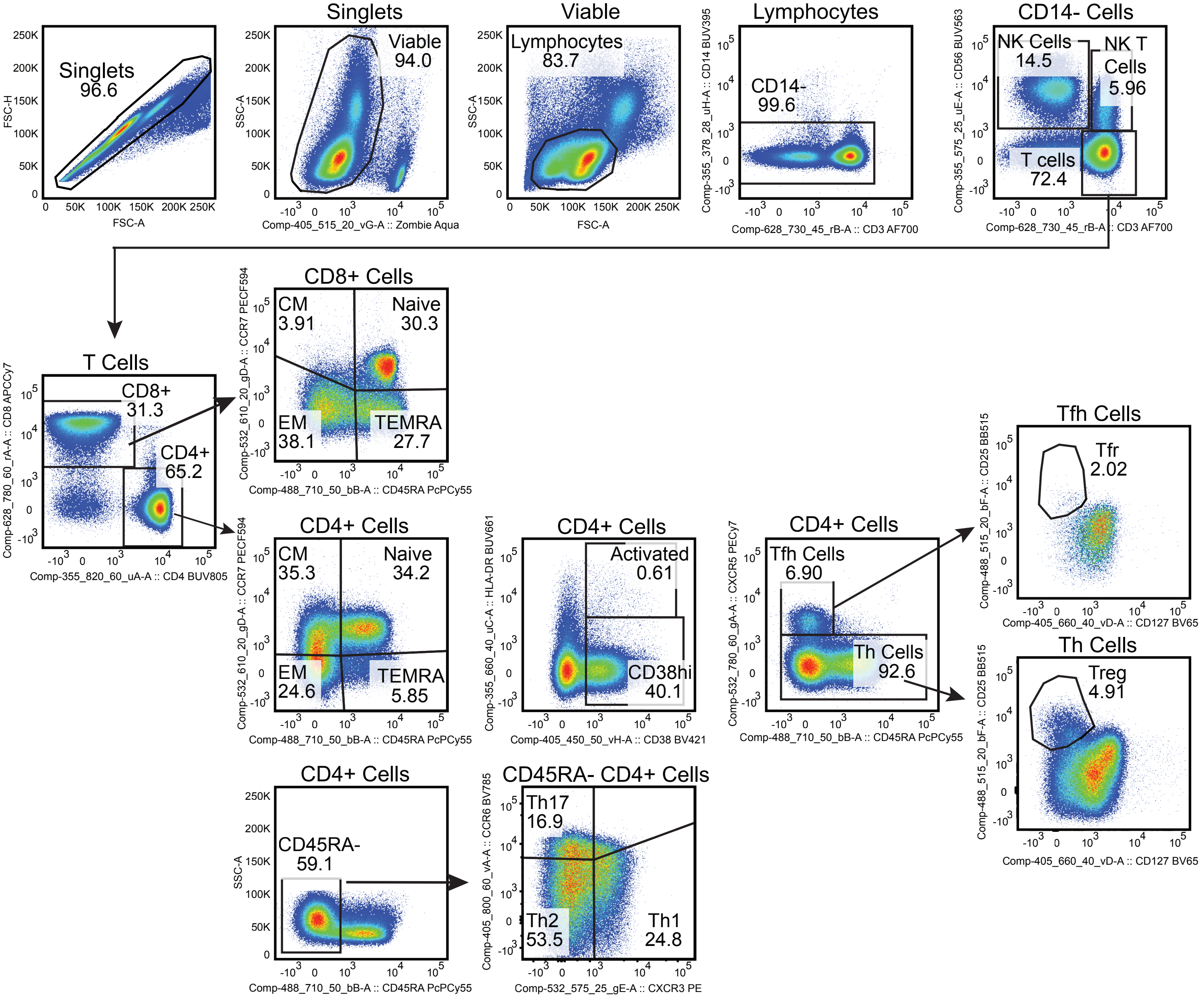

Supplemental Figure 1B

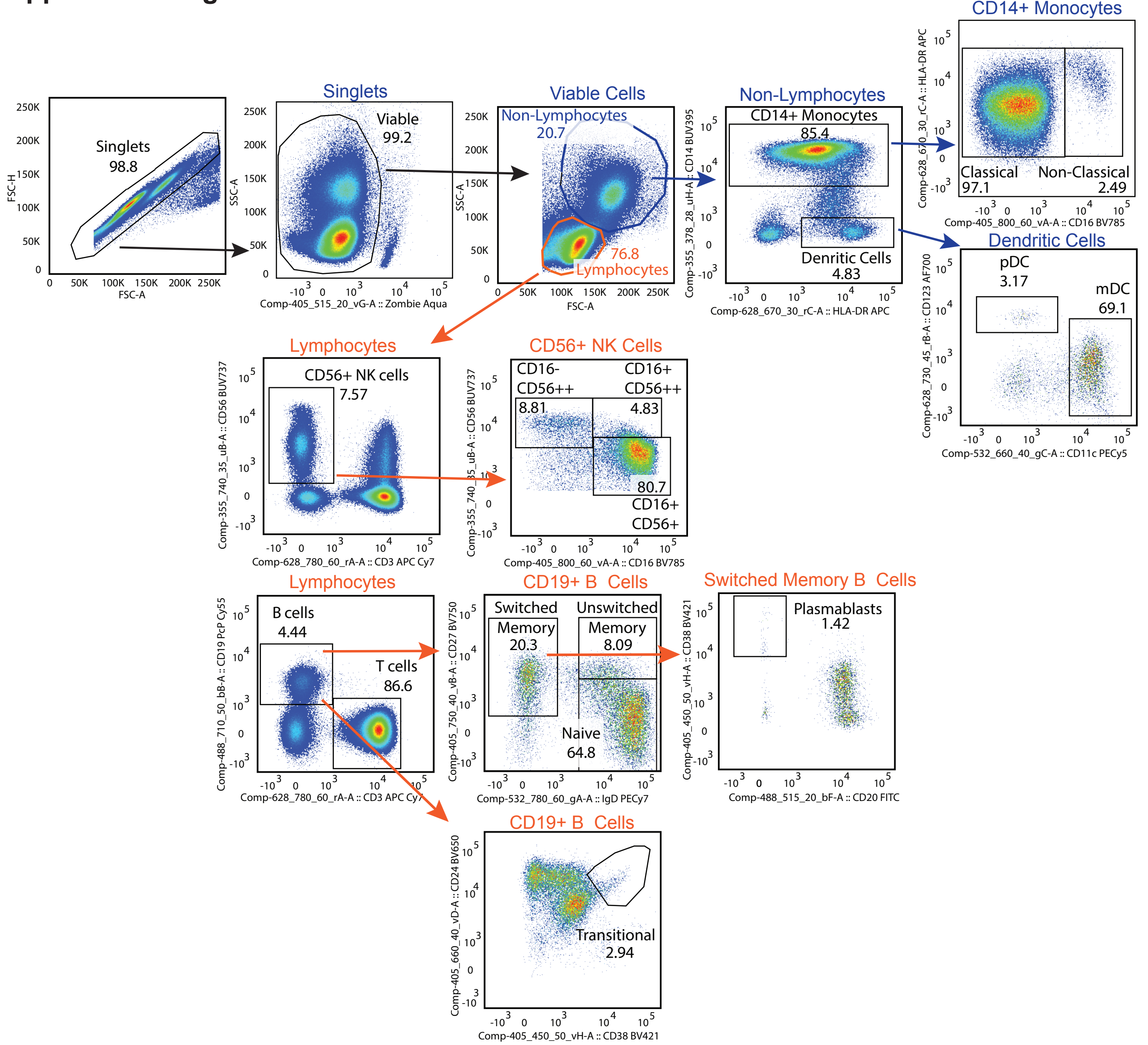

Supplemental Figure 2

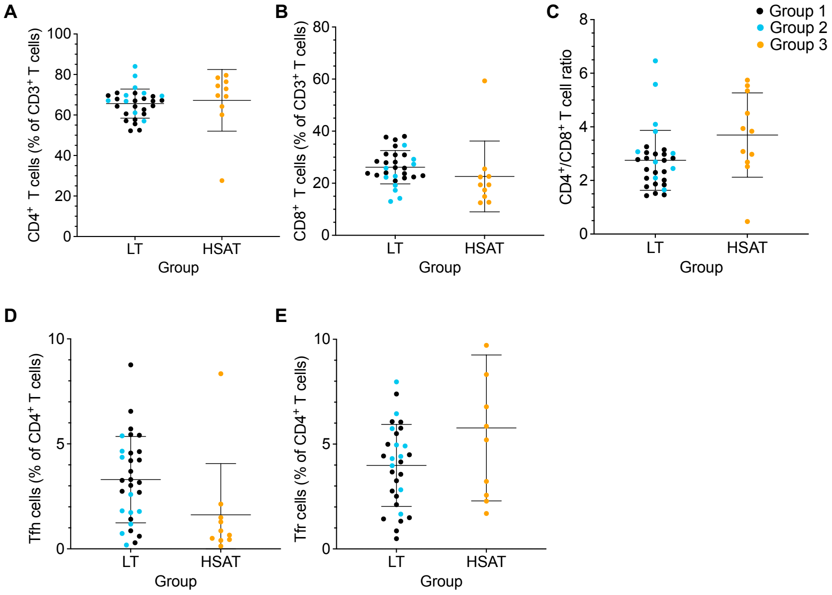

Supplemental Figure 3

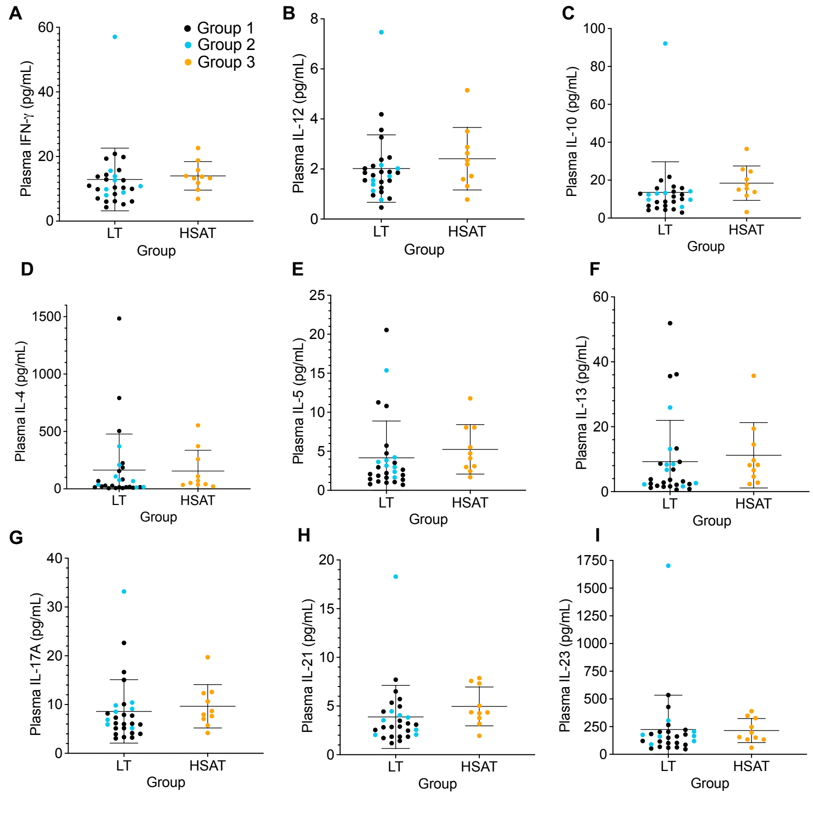

Supplemental Figure 3

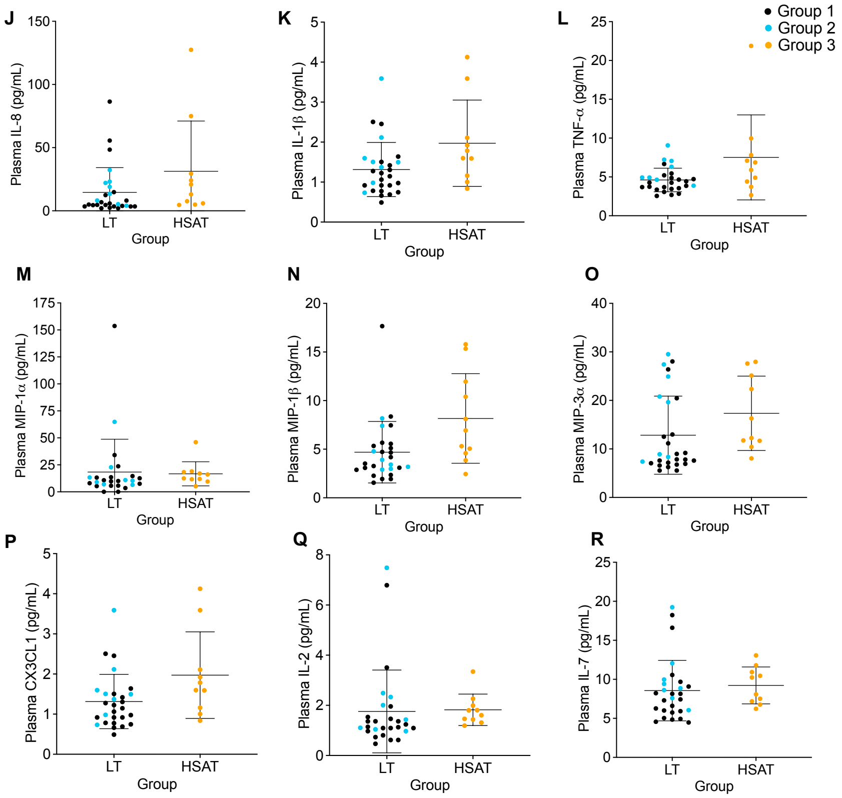

Supplemental Figure 4

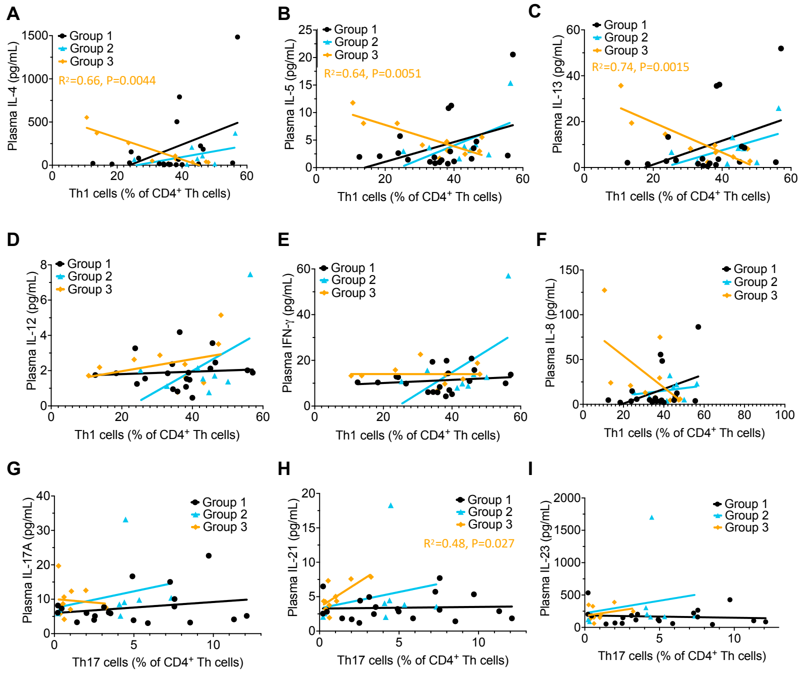

Supplemental Figure 5

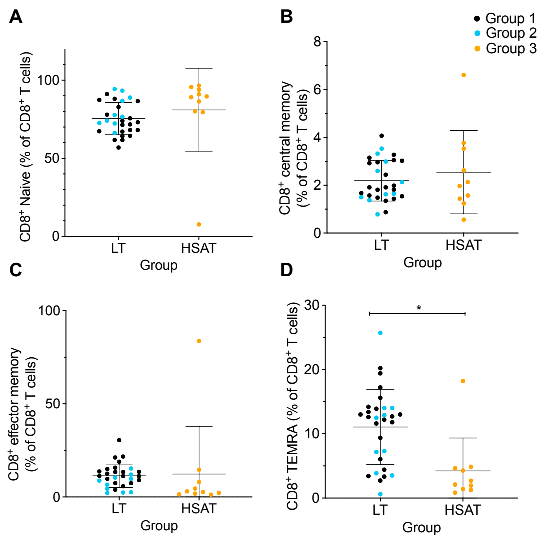

Supplemental Figure 6

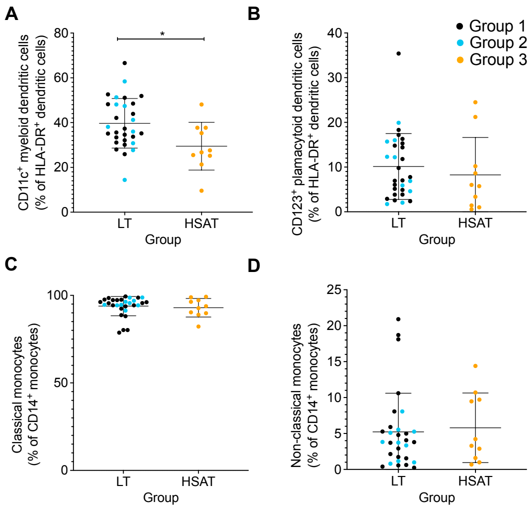

Supplemental Figure 7

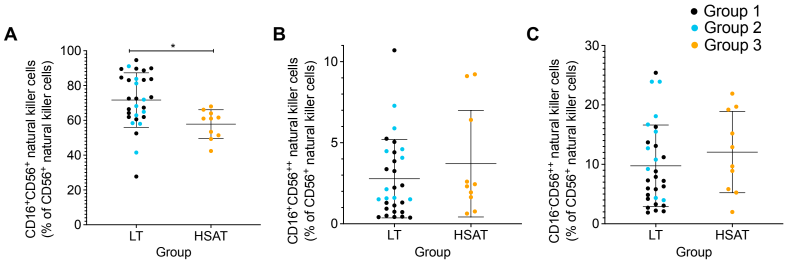

Supplemental Figure 8

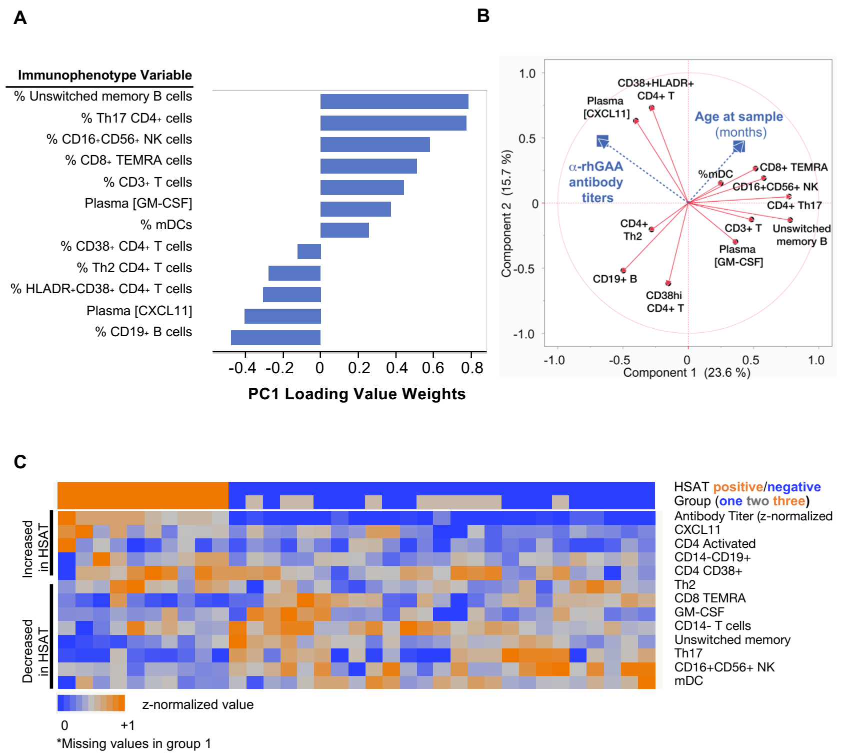

Supplemental Figure 9

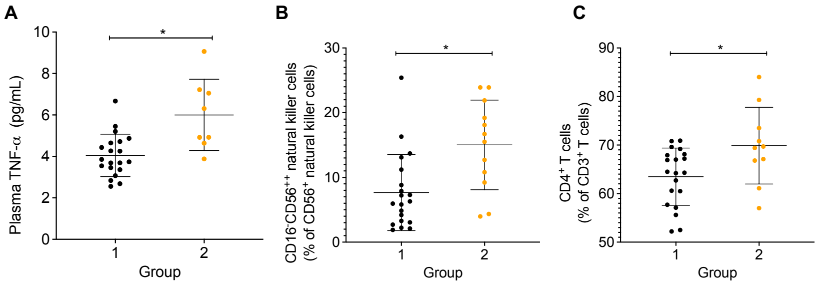

Supplemental Figure 10

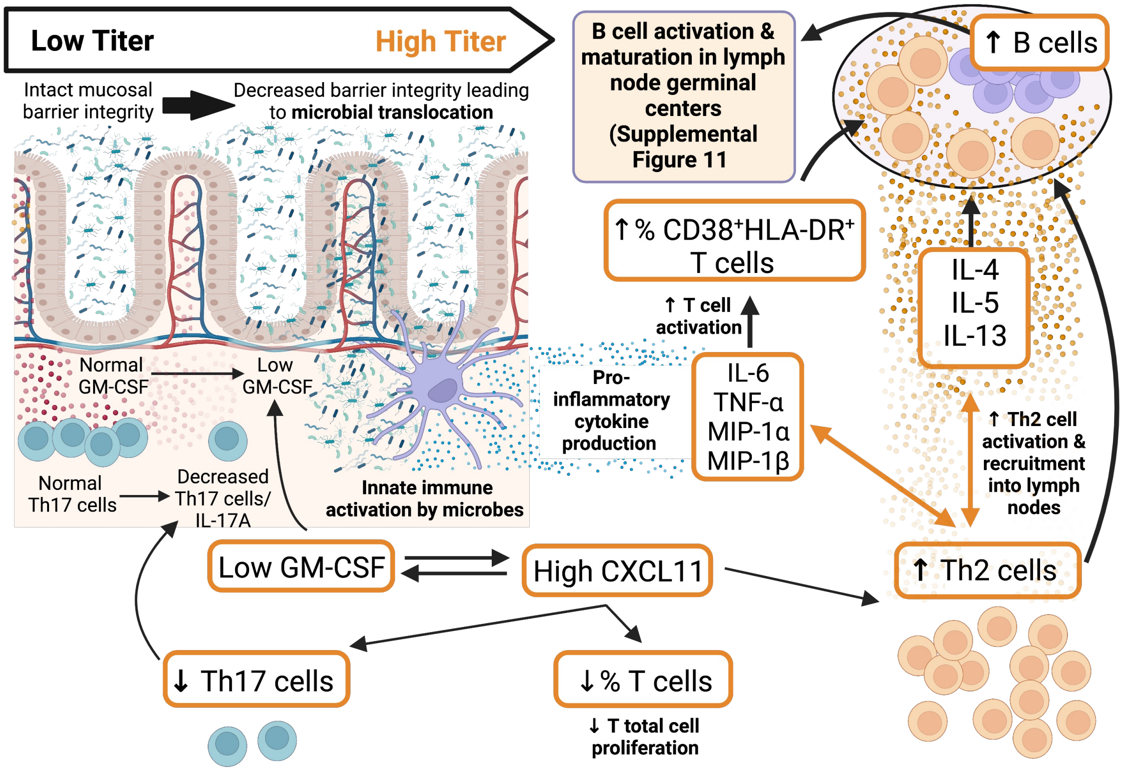

Supplemental Figure 11A

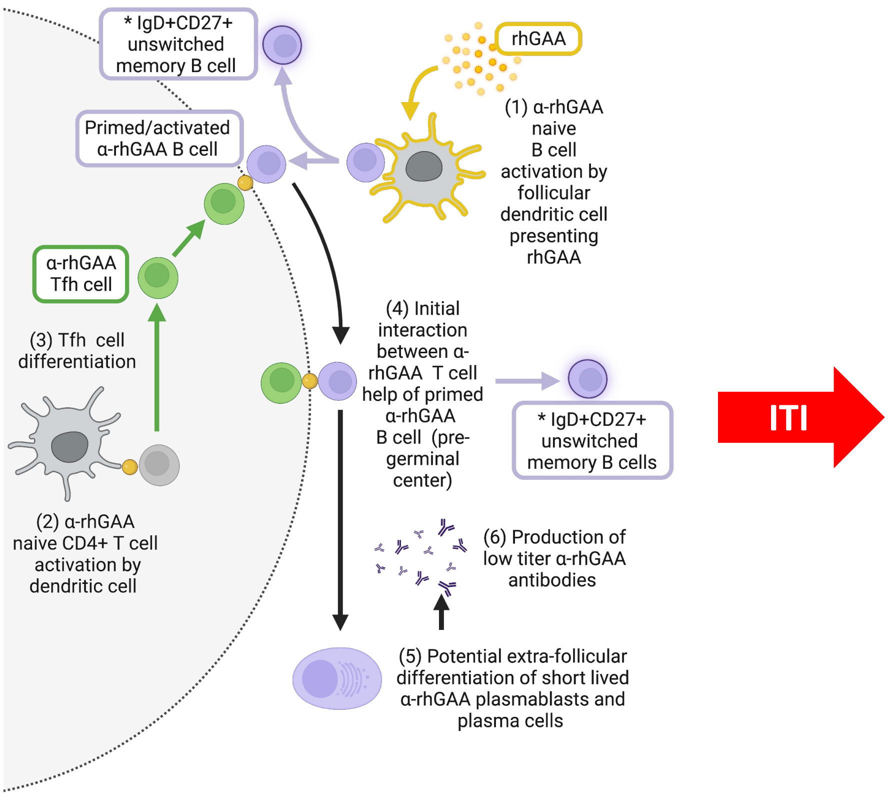

Supplemental Figure 11B

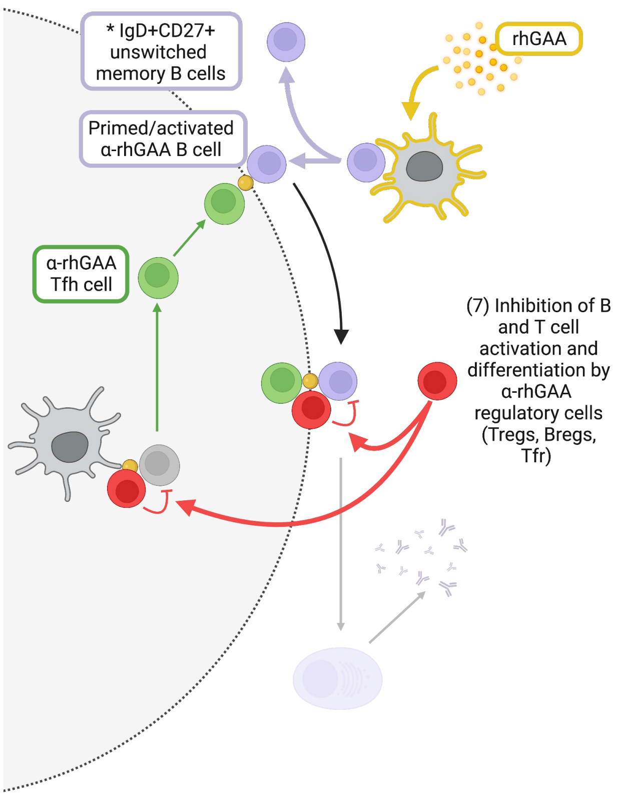

Supplemental Figure 11C

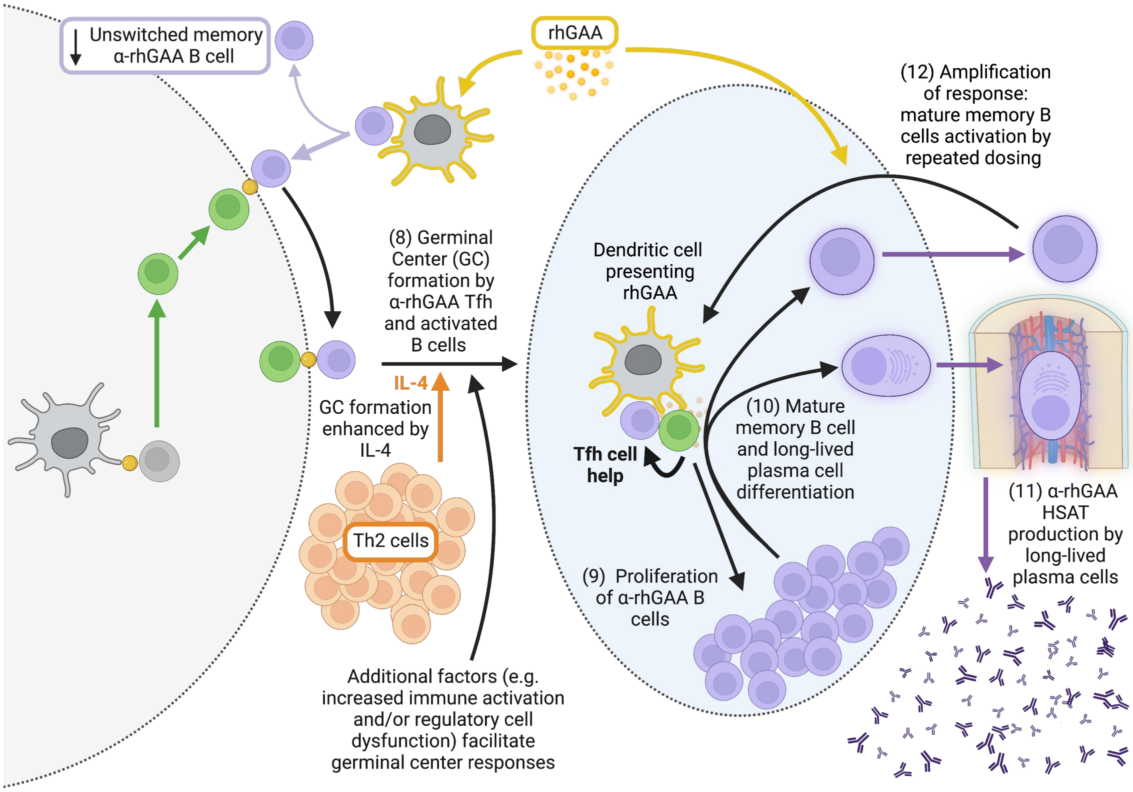

Supplemental Figure 12

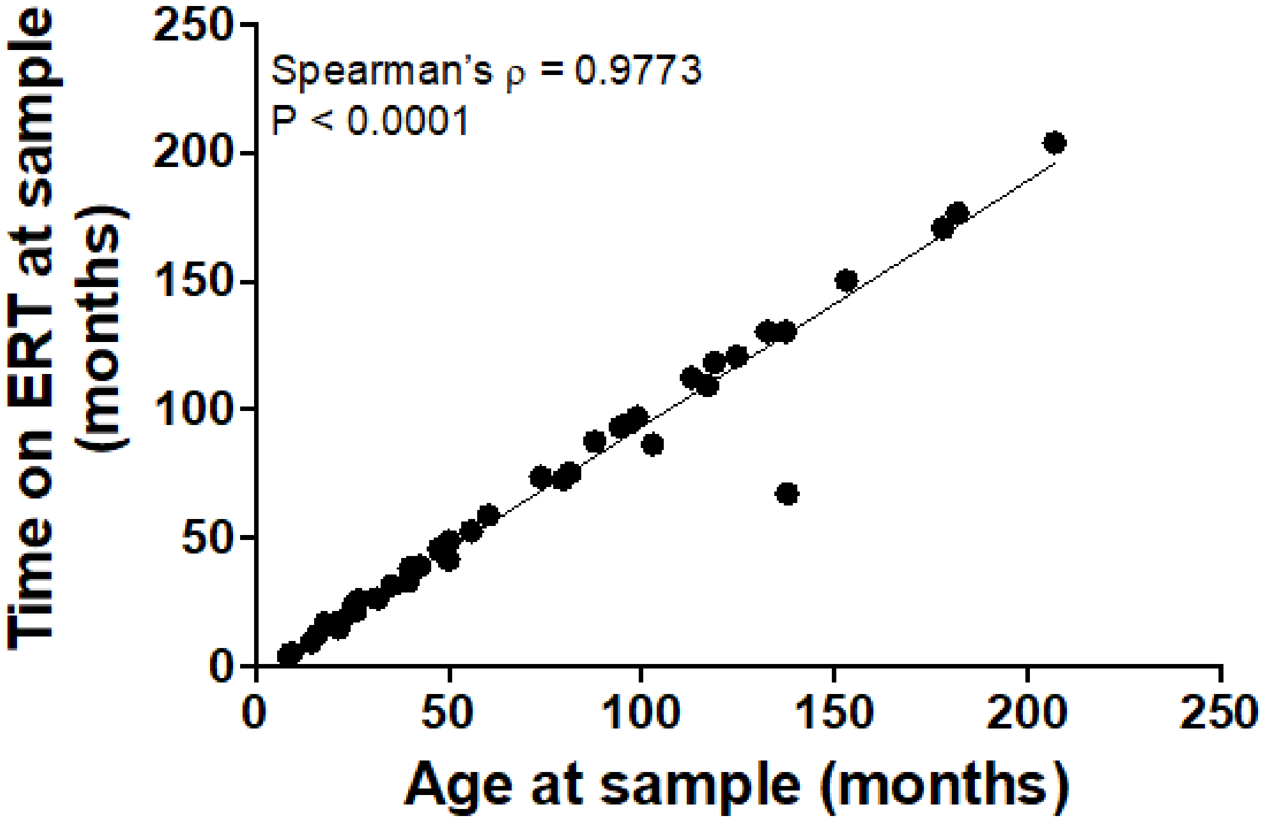

Supplemental Table 1. Flow Cytometry Antibodies

Antibody	Fluor	Supplier	Cat#	Clone
TIGIT	APC	Biolegend	372706	A15153G
CD3	AlexaFluor 700	Biolegend	344822	SK7
CD8	APC-Cy7	Biolegend	344714	SK1
CD25	BB515	BD	564467	2A3
CD45RA	PerCP-Cy5.5	Biolegend	304122	HI100
CD38	BV421	Biolegend	303526	HIT2
Zombie	Aqua Live/Dead	Biolegend		
CCR4	BV605	Biolegend	359418	L291H4
CD127	BV650	Biolegend	351325	A019D5
PD-1	BV750	Biolegend	329966	EH12.2H7
CCR6	BV785	Biolegend	353422	G034E3
CXCR3	PE	BD	557185	1C6/CXCR3
CCR7	PE-CF594	BD	562381	150503
CD19	PE-Cy5	Biolegend	302210	HIB19
CXCR5	PE-Cy7	Biolegend	356924	J252D4
CD14	BUV395	BD	563561	MOP9
CD56	BUV563	BD	612928	NCAM16.2
HLA-DR	BUV661	BD	612981	G46-6
CD39	BUV737	BD	612852	TU66
CD4	BUV805	BD	612887	SK3
CD20	FITC	Biolegend	302350	2H7
CD19	PerCP-Cy5.5	Biolegend	302230	HIB19
CD38	BV421	Biolegend	356618	HB-7
Zombie	Aqua	Biolegend		
CD24	BV650	BD	563720	ML5
CD27	BV750	Biolegend	302850	O323
CD16	BV785	Biolegend	302046	3G8
CD14	BUV395	BD	563561	MOP9
CD56	BUV737	BD	612766	NCAM16.2
HLA-DR	APC	Biolegend	307610	L243
CD123	AlexaFluor 700	Biolegend	306040	6H6
CD3	APC-Cy7	Biolegend	344818	SK7
CD138	PE	Biolegend	356504	MI15
CD11c	PE-Cy5	BD	551077	B-ly6
IgD	PE-Cy7	Biolegend	348210	IA6-2
